# Background trade winds and climate sensitivity control the eastern equatorial Pacific warming pattern

**DOI:** 10.1126/sciadv.aec5746

**Published:** 2026-09-30

**Authors:** Jacob Stuivenvolt-Allen, Alexey V. Fedorov

**Affiliations:** ^1^ Research Applications Laboratory, National Center for Atmospheric Research, Boulder, CO 80303, USA.; ^2^ Department of Earth and Planetary Science, Yale University, New Haven, CT 06516, USA.

## Abstract

Climate models robustly project enhanced warming in the eastern equatorial Pacific (EEP) by the late 21st century, producing a mean El Niño–like pattern that strongly influences atmospheric circulation and El Niño–Southern Oscillation dynamics. Here, we show that the magnitude of this pattern depends primarily on the model’s strength of background Pacific trade winds and the effective climate sensitivity (EffCS), which together explain ∼74% of the intermodel variance in future EEP warming. Perturbation experiments reveal the underlying mechanism: Stronger trade winds produce larger east-west contrasts in sea surface temperature (SST) and low-cloud cover. Under radiative forcing, this amplifies positive low-cloud and Bjerknes feedbacks, leading to a greater reduction in the east-west SST gradient. These results provide an emergent constraint on future changes in the zonal SST gradient. Specifically, an observation-based easterly wind stress of 0.043 newtons per square meter and an EffCS of 3.0°C yields an estimated reduction in the zonal SST gradient of 0.73 under SSP5-8.5.

## INTRODUCTION

Future climate projections critically depend on the evolution of tropical Pacific sea surface temperature (SST) patterns (*1*). In the present climate, the Pacific Walker Circulation (PWC) and easterly trade winds sustain a strong zonal SST gradient along the equator, with warm waters in the western Pacific and colder eastern equatorial Pacific (EEP) due to upwelling across a shallow thermocline. Persistent stratocumulus clouds over the eastern part of the basin also help maintain the east-west temperature contrast. In turn, this temperature contrast sustains the atmospheric PWC. Most climate models project that anthropogenic forcing will weaken the PWC, reduce the zonal SST gradient, and produce an “El Niño–like” enhanced EEP warming pattern (*2*–*4*). However, there is substantial intermodel spread in the magnitude and spatial structure of tropical Pacific warming (*5*), raising the question: How much will the east-west gradient weaken?

The zonal SST gradient shapes internal climate variability, particularly the El Niño–Southern Oscillation (ENSO), mean precipitation patterns and variability, atmospheric teleconnections to the extratropics, and the climate system’s response to external radiative forcing (*6*–*8*). Differential warming alters local radiative feedbacks with enhanced EEP warming being especially influential because it can erode lower-tropospheric stability and stratocumulus cloud cover (*9*–*12*). Reductions in these low clouds amplify local and global warming, and in fact, much of the spread in the climate sensitivity of CMIP6 models [1.8° to 5.6°C for CO_2_ doubling (*13*, *14*)] is believed to arise from tropical Pacific cloud feedbacks (*5*, *12*, *15*, *16*). Thus, understanding the SST warming pattern and its future changes is essential in light of its central role in governing climate variability, feedbacks, and teleconnections.

Several mechanisms have been proposed to explain the projected weakening of the PWC and the east-west SST gradient: stronger evaporative cooling in the western Pacific (*17*, *18*), reductions in eastern low clouds (*19*), off-equatorial influences on the equatorial zonal gradient through the subtropical cells, tropical thermocline stratification, and Southern Ocean coupling (*20*–*22*), and amplification via the Bjerknes and wind-evaporation-SST feedbacks (*23*, *24*). An energetic and hydrological constraint has also been suggested, whereby latent heating due to tropical precipitation increases faster than radiative cooling, slowing the atmospheric circulation and weakening the PWC (*2*, *25*). This midtropospheric constraint, however, may not directly influence the surface, where ocean-atmosphere coupling dominates (*3*, *26*). Last, the ocean thermostat-like effects and anthropogenic aerosol-induced cooling can temporarily delay the gradient reduction by maintaining cold upwelling in the east (*27*–*29*).

In this study, we investigate whether the future pattern of Pacific SST warming—particularly the reduction in the zonal SST gradient—can be anticipated from a model’s preindustrial or historical mean-state and other model characteristics. Specifically, we focus on the historical strength of the mean Pacific trade winds, quantified by surface wind stress, as a proxy for the strength of the PWC (*30*). We also consider the role of each model’s effective climate sensitivity (EffCS), to account for the differences in the transient response to CO_2_ forcing across 25 CMIP6 models. To isolate the role of mean-state dynamics from confounding factors such as differing EffCS values, we conduct a set of perturbation experiments using the Community Earth System Model version 2 (CESM2). By systematically altering the mean trade wind stress and PWC strength before warming in these experiments, we evaluate the physical mechanisms through which these background conditions influence the resulting pattern of future SST warming.

## RESULTS

### Projected changes in the tropical Pacific across CMIP6 models

Figure 1A shows the multimodel ensemble mean of SST and zonal wind stress from the CMIP6 Historical simulations (1900 to 1999). The climatological pattern features strong easterly trade winds across the equatorial Pacific, a well-developed cold tongue in the east, and a pronounced warm pool in the western Pacific.

**Fig. 1.**
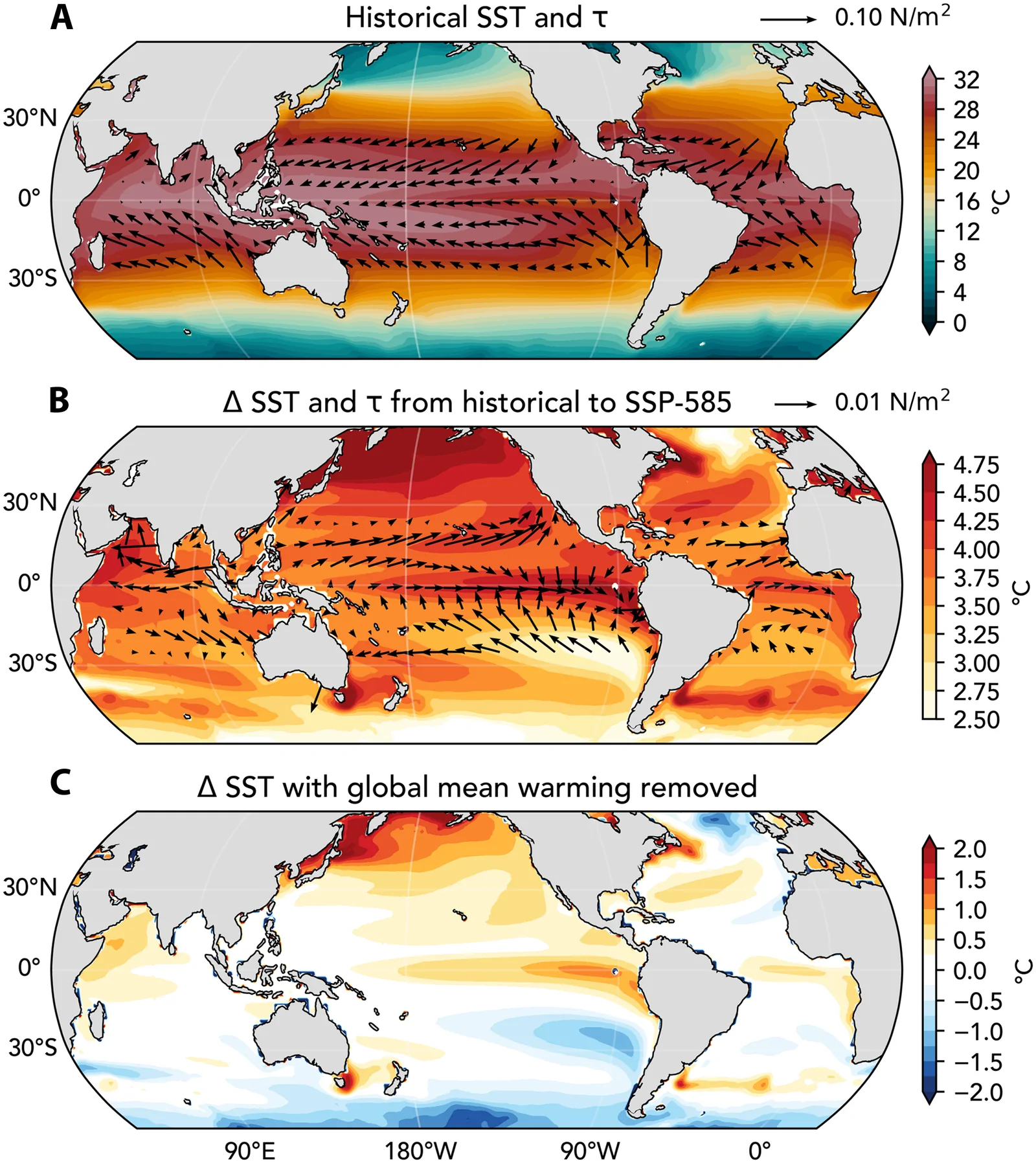
Model simulated historical SST and wind stress along with their projected change. (**A**) The multimodel ensemble mean climatology of SST (shading) and wind stress (vectors) for 25 CMIP6 models averaged from 1900 through 1999. (**B**) The projected difference of these fields from the Historical scenario (1900 to 1999) to SSP5-8.5 (2050 to 2099). (**C**) The SST change with the global mean removed, highlighting the enhanced warming in the EEP.

By the end of the 21st century (2050 to 2099, SSP5-8.5 scenario), substantial changes to these climatological fields are evident (Fig. 1B). The trade winds weaken, particularly in the central and eastern Pacific, leading to anomalous low-level wind convergence in the eastern equatorial region. Figure 1C shows the projected SST change with the tropical-mean warming removed to isolate spatial pattern differences. While we focus on the SSP5-8.5 minus Historical difference to illustrate these projected changes, similar patterns emerge across other forcing scenarios and individual models (*31*–*33*).

Using the CMIP6 models listed in table S1, we evaluate the equatorial Pacific zonal wind stress (area averaged from 5°S to 5°N and 150°W to 100°W) in each model’s preindustrial control (piControl) simulation, which is a proxy for the strength of the PWC. We then relate this metric to the change in the equatorial SST gradient from the Historical scenario (1900 to 1999) to the SSP5-8.5 scenario (2050 to 2099). The zonal SST gradient is calculated by subtracting the western Pacific warm pool SST (5°S to 5°N, 80° to 140°E) from the EEP SST (5°S to 5°N, 140° to 80°W), and a positive change indicates a weakening of the gradient. A clear relationship emerges: Models with stronger climatological trade winds in the Historical scenario exhibit a greater reduction in the zonal SST gradient under forcing (Fig. 2A).

**Fig. 2.**
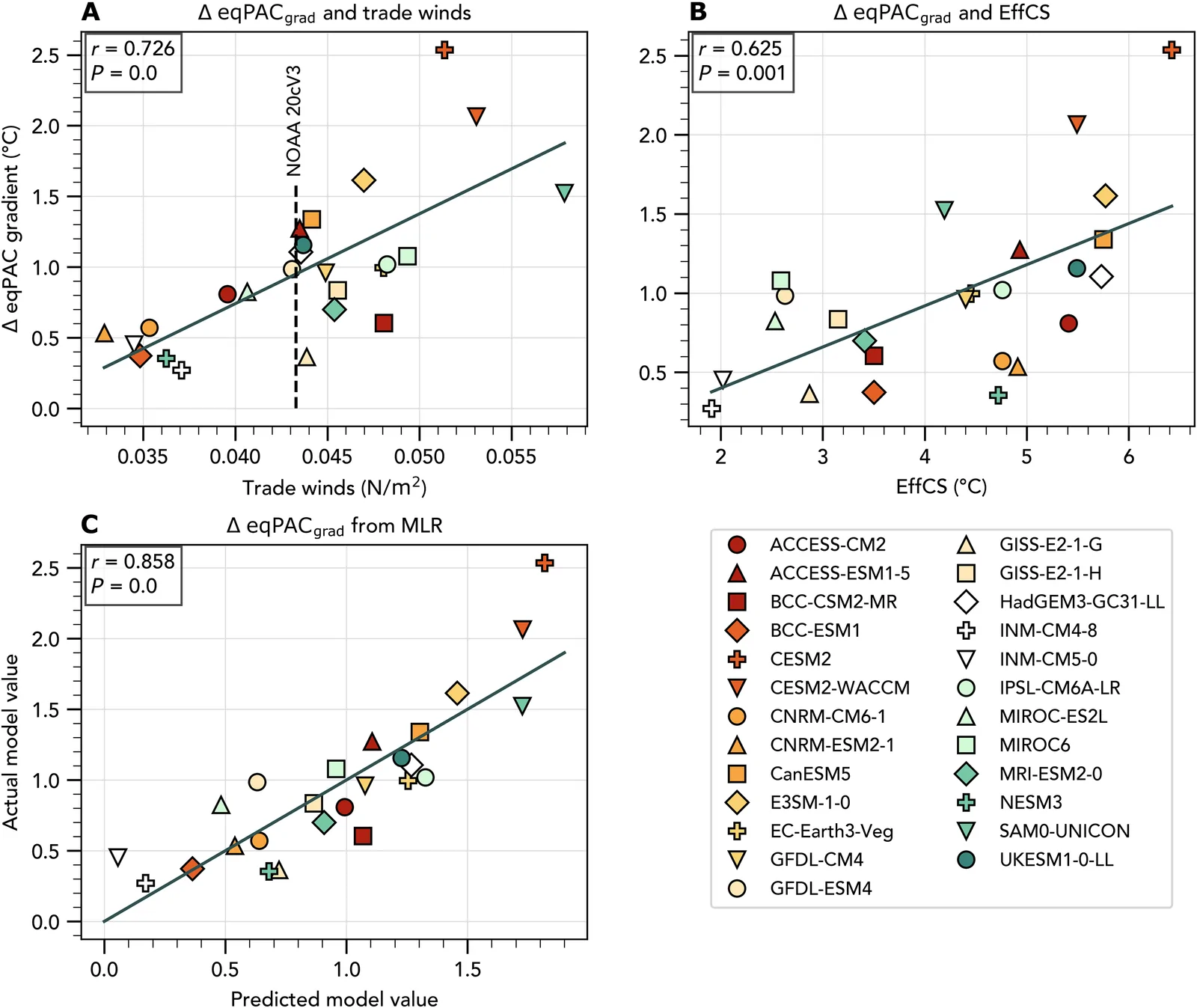
Projected changes in the equatorial Pacific zonal SST gradient as a function of background trade wind strength and EffCS. (**A**) Scatterplot of the mean trade winds in the Historical scenario (1900 to 1999) compared with the model’s projected change in the equatorial Pacific SST gradient under SSP5-8.5 (2050 to 2099). (**B**) Projected change in the SST gradient versus each model’s EffCS. (**C**) EffCS and the background trade wind strength combined as predictors of the future change in the equatorial SST gradient using multiple linear regression. Correlation coefficients from the linear fits are provided in the top-left of each subplot. The mean trade wind strength from NOAA 20th Century Reanalysis Version 3 (*52*) is indicated by the vertical line in (A) to provide an observations-based estimate for 1900 to 1999.

Despite this robust relationship, intermodel spread remains. This residual variability may arise from a number of factors including differences in model physics and parameterizations, especially those related to shortwave-cloud feedbacks (*34*). To further constrain this spread, we assess whether models’ EffCS can help explain the projected changes in SST gradient. The rationale is straightforward: This parameter reflects key physical processes operating in the climate system, and models with higher EffCS experience stronger global warming and may consequently exhibit larger regional responses in the equatorial Pacific.

The projected change in the equatorial SST gradient is found to significantly correlate with EffCS (Fig. 2B). EffCS values for the 25 CMIP6 models are adopted from Dong *et al.* (*12*), who derived them from global mean temperature trajectories over the 150-year period following CO_2_ quadrupling. These values reflect an effective or transient sensitivity, not the equilibrium climate sensitivity, which may require multimillennial simulations to estimate (*35*). Nonetheless, the projected change in the equatorial SST gradient is linked to EffCS values (Fig. 2B).

Last, we assess the predictive power of combining both mean-state trade wind strength and EffCS in explaining the SST gradient response across models. A multiple linear regression using historical mean trade wind strength and EffCS as predictors accounts for ∼74% of the intermodel variance in the SST gradient change following CO_2_ increase. We stress that the magnitude of the mean trade wind stress and each model’s EffCS values are uncorrelated and independent predictors [correlation coefficient (*r*) = 0.24 and a negligible variance inflation factor of 1.06] (fig. S2).

Next, we compare the projected change in the zonal SST gradient with the mean trade wind strength from the piControl runs and models’ EffCS in CMIP6 under the CO2 quadrupling scenario (fig. S1). Our analysis produces analogous results, supporting the robustness of the relationship between SST gradient changes, the mean trade winds, and EffCS.

We repeat the analysis displayed in Fig. 2A using the CESM2 large ensemble—a set of 100 simulations that are initialized with minor temperature perturbations and at different states of the Atlantic Meridional Overturning Circulation to elicit 100 realizations with historical external forcings and extended into the 21st century under SSP3-7.0 (*36*, *37*). Even within the large ensemble, the projected change in the zonal SST gradient for the 21st century correlates well with the strength of the trade winds in the Historical scenario (1900 to 1999) (fig. S3A), and this is despite the relatively narrow range of mean trade wind variation across the ensemble compared to CMIP6 models.

The climate sensitivity of these 100 ensemble members should be quite similar, as differences in the global mean temperature response to CO_2_ increase within the CESM2 large ensemble are expected to come from internal variability alone. Figure S3B shows a comparison between end-of-century (2050 to 2099) global mean temperature change (used as a proxy for the global temperature sensitivity to radiative forcing) in a given CESM2 large-ensemble member and its respective change in the tropical Pacific SST gradient. These metrics are not related, highlighting that internal variability in the global mean temperature response to radiative forcing does not substantially affect century-long changes in the east-west SST gradient.

### Warming experiments under modified mean trade wind strengths within CESM2

To further investigate the role of tropical Pacific mean-state winds in shaping future SST responses, we conduct a series of global warming experiments with modified mean states using CESM2. In these experiments, we systematically reduce the strength of the background equatorial trade winds by applying momentum flux perturbations to create altered mean states, which are then integrated for 100 years to reach a quasi-equilibrium. Subsequently, we quadruple atmospheric CO_2_ and continue simulations for another 150 years. These experiments, described in detail in Materials and Methods, are designed to isolate the influence of the mean trade winds and SST gradient on the climate response within a single model, while maintaining nearly the same EffCS.

Figure S4 illustrates the zonal surface momentum flux anomalies applied in these experiments. The perturbation—a two-dimensional Gaussian anomaly centered on 120°W and the equator—represents westerly wind-stress anomalies superimposed on the climatological easterlies. This perturbation is applied at every model timestep, maintaining a stable but weakened trade wind stress throughout the simulation. Given that CESM2 has one of the strongest climatological trade winds among CMIP6 models (see Fig. 2), we perform two perturbation experiments with trade wind stress reduced by ∼10 and 20% relative to the CESM2 control. These experiments are referred to as the Moderate and Weak Trades simulations, respectively, while the default CESM2 configuration serves as the Strong Trades simulation.

The impacts of these surface momentum flux adjustments under fixed preindustrial CO_2_ values (284 ppm) are evident in the mean-state zonal wind stress and SST fields (Fig. 3, A and B). The Moderate Trades experiment produces a clear warming of the EEP, reducing the mean zonal SST gradient by ∼0.75°C, without substantially altering the global mean temperature. The resulting pattern resembles a weak mean El Niño–like state, with enhanced warming in the cold tongue region. In the Weak Trades experiment, the reduction in trade wind strength is even more pronounced, and the mean-state zonal SST gradient is further diminished by nearly 1.25°C compared to the Strong Trades simulation. The coupling between the SST gradient and the overlying atmospheric circulation also implies that the perturbation experiments have a weaker PWC.

**Fig. 3.**
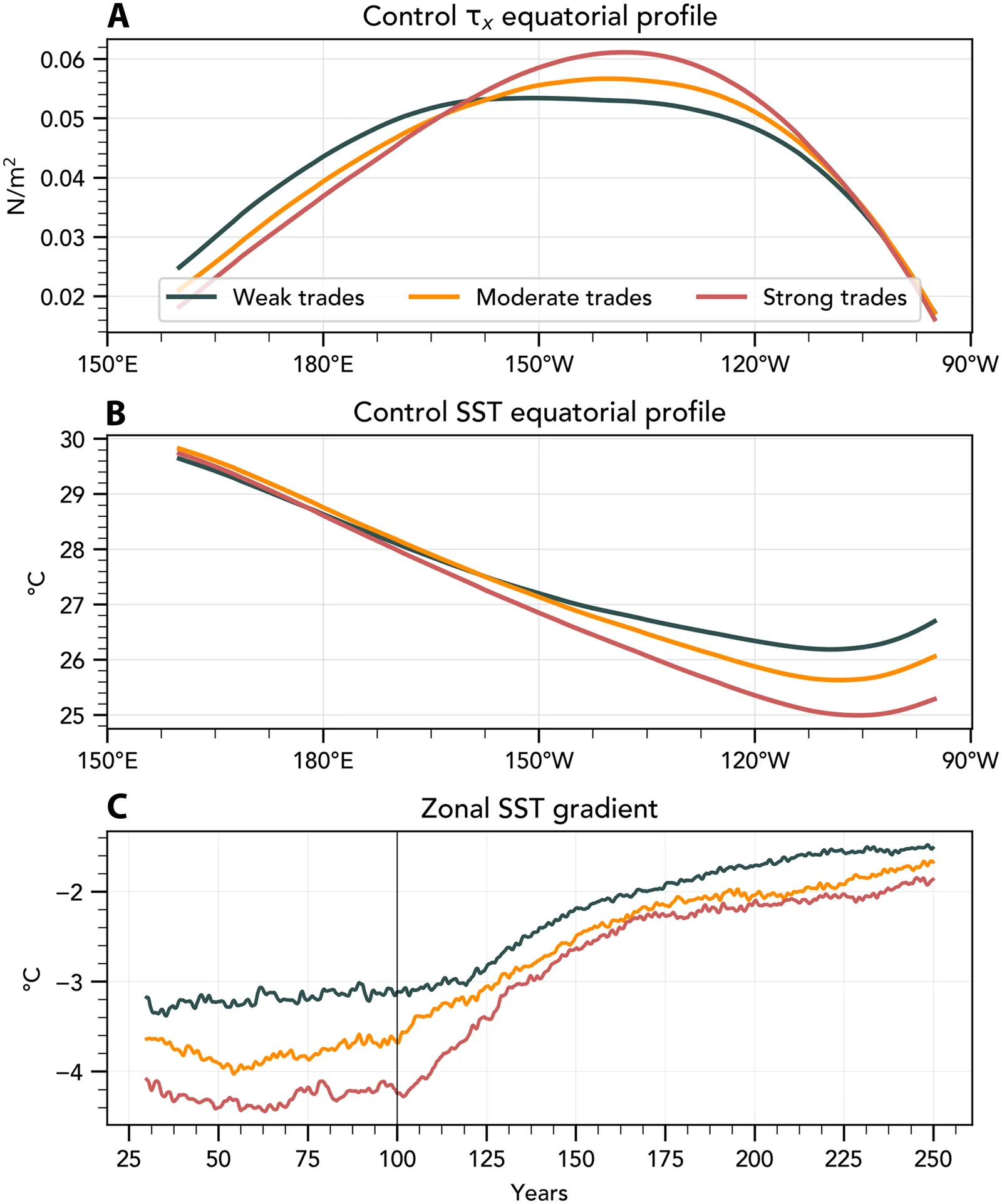
Results of the modified mean-state experiments. Equatorial profiles (averaged latitudinally from 5°S to 5°N) of $\tau_{x}$ (**A**) and SST (**B**) for the first 100 years of each perturbation experiment (preindustrial CO_2_). (**C**) Evolution of the equatorial Pacific SST gradient in the experiments. CO_2_ concentration is quadrupled after year 100, marked by the vertical black line. A 30-year moving average applied to smooth out internal variability.

After allowing the three simulations to evolve under preindustrial conditions for 100 years, we impose an abrupt quadrupling of atmospheric CO_2_ (to 1136 ppm) and continue each simulation. Figure 3C shows the subsequent evolution of the equatorial Pacific SST gradient over time, computed as the area-averaged SST difference between the western Pacific Warm Pool and the eastern Pacific Cold Tongue.

Substantial differences in the SST evolution between experiments emerge within several decades after CO_2_ forcing. As shown in Figs. 3C and 4, the reduction of the zonal SST gradient and the enhanced EEP warming pattern that characterizes the Pacific responses are critically dependent on the initial trade wind strength. In the Strong Trades experiment, this pattern emerges rapidly after the onset of forcing, with a pronounced warming maximum in the eastern Pacific. Reductions in the low-cloud fraction and zonal wind-stress changes are complementary to the underlying SST changes. The reduction in low-cloud cover (areas that receive a 25% reduction in low-cloud fraction are stippled) is quite muted for the Weak Trades experiment, but the Strong Trades experiment experiences a widespread reduction of the stratocumulus deck (Fig. 4).

**Fig. 4.**
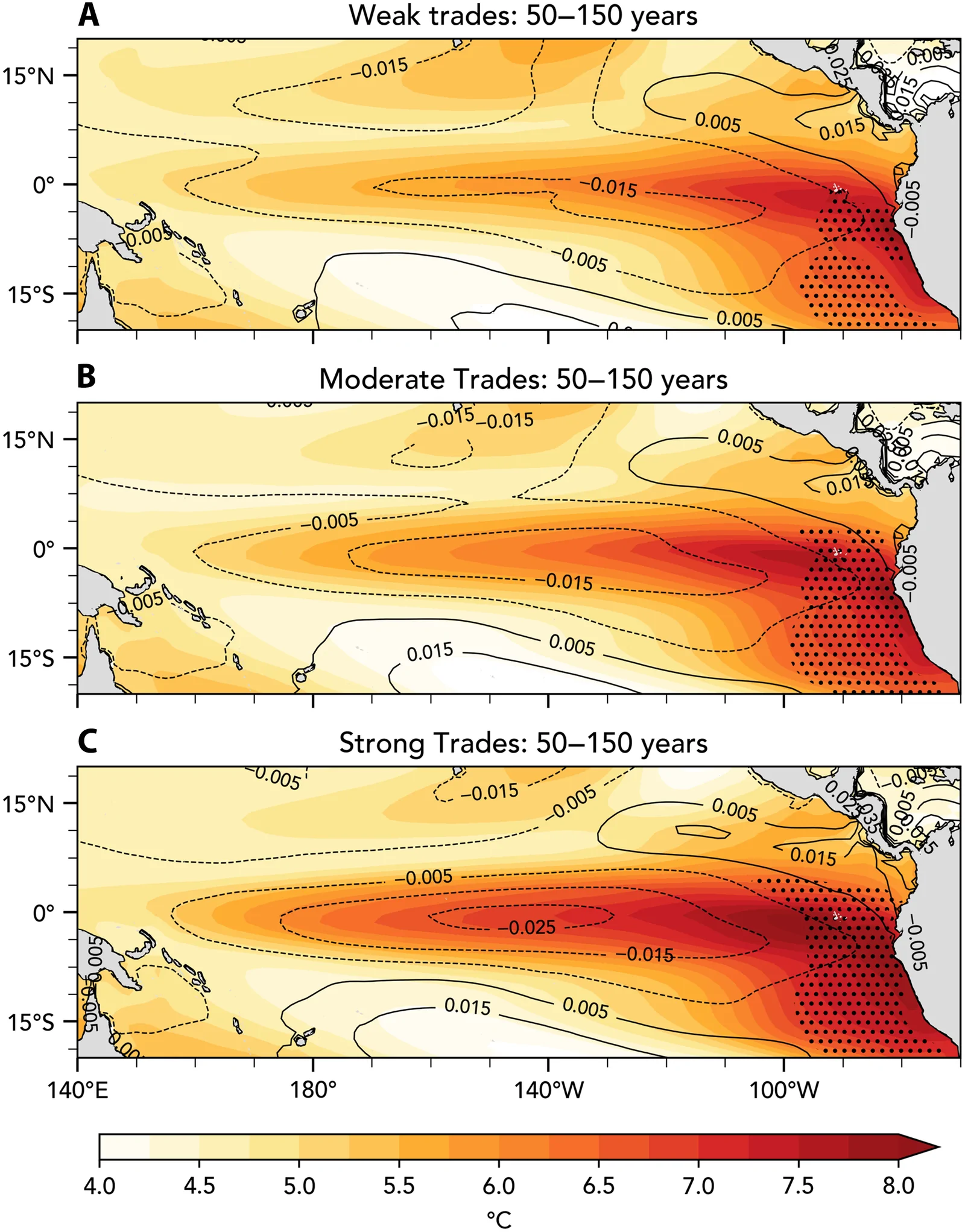
Warming patterns in the modified mean-state experiments under 4×CO_2_. Anomalies in SST (color shading), $\tau_{x}$ (contours), and low-cloud fraction (area experiencing a 25% reduction is stippled) after quadrupling CO_2_ relative to the first 100 years of the modified mean-state simulations for the (**A**) Weak, (**B**) Moderate, and (**C**) Strong Trades experiments. Each displayed variable represents the mean over the final 100 years of the simulation.

Although all three experiments ultimately develop an EEP warming pattern by the end of the simulations, the time of emergence and spatial extent of the EEP warming vary substantially. In the Weak Trades experiment, the zonal SST gradient remains relatively weak throughout, whereas the Strong Trades simulation shows a more pronounced weakening of the gradient, dominated by preferential warming of the eastern basin associated with a greater loss of low-cloud cover (Fig. 4C).

The shortwave and low-cloud feedbacks are strongest in the Strong Trades experiment, where the large initial zonal SST gradient supports extensive stratocumulus decks that are eroded under warming (Figs. 4C and 5). Net surface heat flux into the EEP is greatest in the Strong Trades simulation and weakest in the Weak Trades simulation (figs. S5 and S6). In all three experiments, the western Pacific warm pool experiences a marked reduction in incoming shortwave radiation, consistent with enhanced convection and cloud development accompanying warmer SSTs. Critically, these shortwave flux changes act to erode the zonal SST gradient more strongly for the Strong Trades case (also shown in fig. S7).

**Fig. 5.**
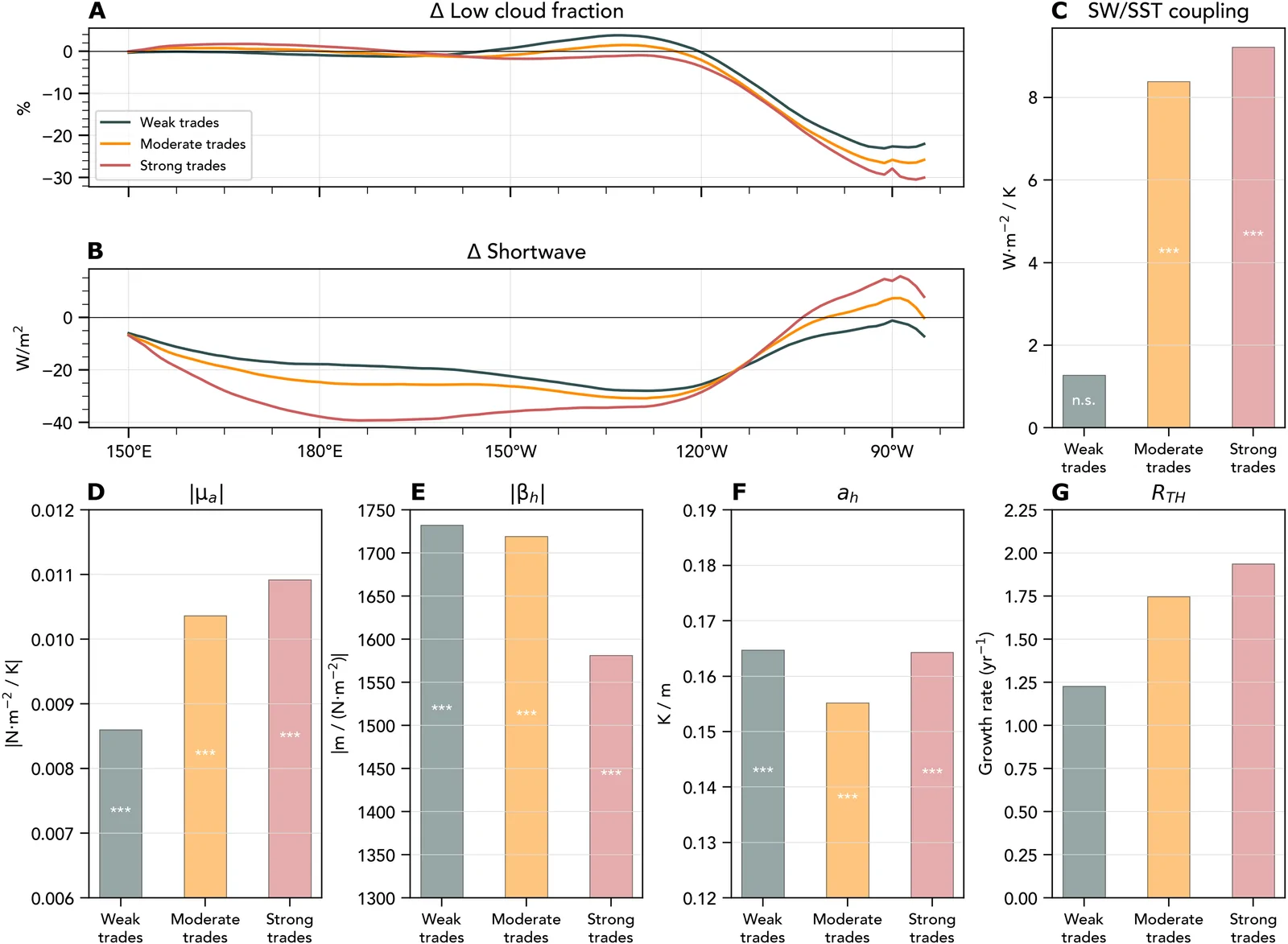
Equatorial Pacific changes in low cloud cover and shortwave flux under 4×CO_2_, and the strength of the ocean-atmosphere coupling in the modified wind-stress experiments. (**A**) Changes in low cloud fraction and (**B**) net surface shortwave radiation, averaged over 5°S to 5°N as a function of longitude, in response to 4×CO_2_. (**C**) Shortwave flux and SST coupling, estimated from the regression slope of net surface shortwave flux onto SST over the EEP (120°W to 80°W, 5°S to 5°N). Dynamical ocean-atmosphere coupling coefficients (Materials and Methods): (**D**) atmospheric coupling $|\mu_{a}|$, (**E**) ocean dynamical coupling $|\beta_{h}|$, and (**F**) subsurface temperature coupling $a_{h}$. (**G**) The Bjerknes feedback, $R_{\text{TH}}$ (Eq. 5, Materials and Methods). All regressions use data with a 5-year rolling mean applied, with *P* values computed assuming 20 effective degrees of freedom. Significance levels: ***P < 0.001; n.s., not significant.

The strength of the shortwave-flux feedback is shown in Fig. 5C, estimated as the regression slope of eastern tropical Pacific net surface shortwave flux onto SST (120°W to 80°W, 5°S to 5°N) using 5-year rolling means to isolate the slow, forced response from interannual ENSO variability. This coupling increases sharply with trade wind strength (Fig. 5C): The regression slope is +1.27 W m^−2^ K^−1^ in Weak Trades (not significant), compared to +8.38 in Moderate Trades and +9.22 in Strong Trades (both *P* < 0.001). In the Moderate and Strong Trades experiments, extensive stratocumulus decks over the eastern Pacific thin and break up as SSTs warm, producing a strong positive shortwave feedback that reinforces surface warming (*19*, *38*). In the Weak Trades experiment, however, this direct relationship breaks down: The weak zonal SST gradient supports fewer low clouds to begin with (Fig. 4A and fig. S8), and the warmer eastern Pacific is instead more strongly coupled to convection, which reduces rather than enhances incoming shortwave radiation. The result is a different radiative regime in which the positive shortwave feedback that amplifies gradient changes in the other experiments is disrupted (fig. S9A).

Another factor that can amplify the EEP pattern in the Strong Trades experiment is the Bjerknes feedback, whereby SST anomalies generate wind stress anomalies that, in turn, amplify the SST anomalies. Here, we compute the thermocline component of this feedback (Materials and Methods). Following (*39*), which describes the Bjerknes Stability Index in detail, we decompose the coupling chain into three components: how strongly the atmosphere responds to SST anomalies (atmospheric coupling, $\mu_{a}$), how efficiently wind stress anomalies tilt the equatorial thermocline (ocean dynamical coupling, $\beta_{h}$), and how strongly subsurface temperatures in the eastern Pacific respond to thermocline depth changes (subsurface temperature coupling, $a_{h}$; see Fig. 5, D to F). Each coupling coefficient is estimated as a regression slope from the control climate of each experiment. Unlike (*39*), we again apply a 5-year rolling mean to the original data to isolate feedbacks acting on longer timescales rather than interannual timescales (Fig. 5, D to F, fig. S9, and Eqs. 2 to 4).

To obtain the stability index, the product of these three coupling coefficients is scaled by the mean upwelling ($\bar{w}$) acting over a mixed-layer depth scale (*H*_m_), which incorporates the effects of differences in $\bar{w}$ arising from different trade wind regimes. A closely related term representing SST damping by mean upwelling should also be added (Materials and Methods; Eq. 5). The resulting term, yielding growth rate of SST anomalies, provides a combined measure of the thermocline component of the Bjerknes feedback, which we term *R*_TH_.

We subsequently compare the strength of the Bjerknes feedback, expressed as *R*_TH_, across the first 100 years of our modified wind-stress experiments. We find that *R*_TH_ increases monotonically with trade wind strength–by about 58% from the Weak to Strong Trades (Fig. 5G), whereas the other two coupling coefficients either partially compensate or do not influence this increase (Fig. 5, E and F). Together, these results demonstrate that coupled ocean-atmosphere feedbacks are indeed stronger under strong trade wind regime characterized by a robust east-west SST gradient. Consequently, initial warm anomalies induced in the EEP are amplified more when radiative forcing is applied to a state with a large east-west SST contrast.

## DISCUSSION

This study demonstrates that the magnitude of the projected EEP warming across climate models is highly sensitive to the strength of background trade winds under preindustrial or historical conditions and to the model’s EffCS. Across CMIP6, we find that stronger mean-state easterly trade winds consistently lead to a more pronounced EEP warming response to CO_2_ forcing and a greater reduction in the east-west SST gradient along the equator. Likewise, a larger EffCS leads to greater EEP warming. In contrast, weaker trades and smaller EffCS moderate the warming of the EEP, producing a more zonally uniform SST response.

A number of studies have argued that EffCS values in CMIP6 are, on average, too large, compared with the likely range of 2.5° to 4°C obtained from broader assessments (*40*–*44*). Using our multiple linear regression model, one can estimate the magnitude of the future zonal SST gradient change for this likely EffCS range and across observation-based trade wind strengths (see Materials and Methods). This approach narrows the range of projected gradient reduction in CMIP6 under SSP5-8.5 from about 0.25° to 2.5°C (*20*) to a more moderate 0.16° to 1.12°C.

To translate these relationships into concrete projections, we account for observational uncertainty in trade wind strength using bootstrap resampling of three reanalyses and the TAO array data (see Materials and Methods). The observed 1990 to 2024 mean state from the TAO array falls well within the reanalysis spread, providing confidence in the constraint. It is also worth noting that the trade wind strength determined from a century-long reanalysis product (NOAA 20th Century V3) yields a mean trade wind stress of 0.043 N/m^2^ for 1900 to 1999, close to the TAO observed value. Consequently, under SSP5-8.5, combining this value of mean wind stress (0.043 N/m^2^) with a 3.0°C sensitivity to CO_2_ doubling predicts a zonal SST gradient reduction of 0.73° ± 0.05°C (Fig. 6).

**Fig. 6.**
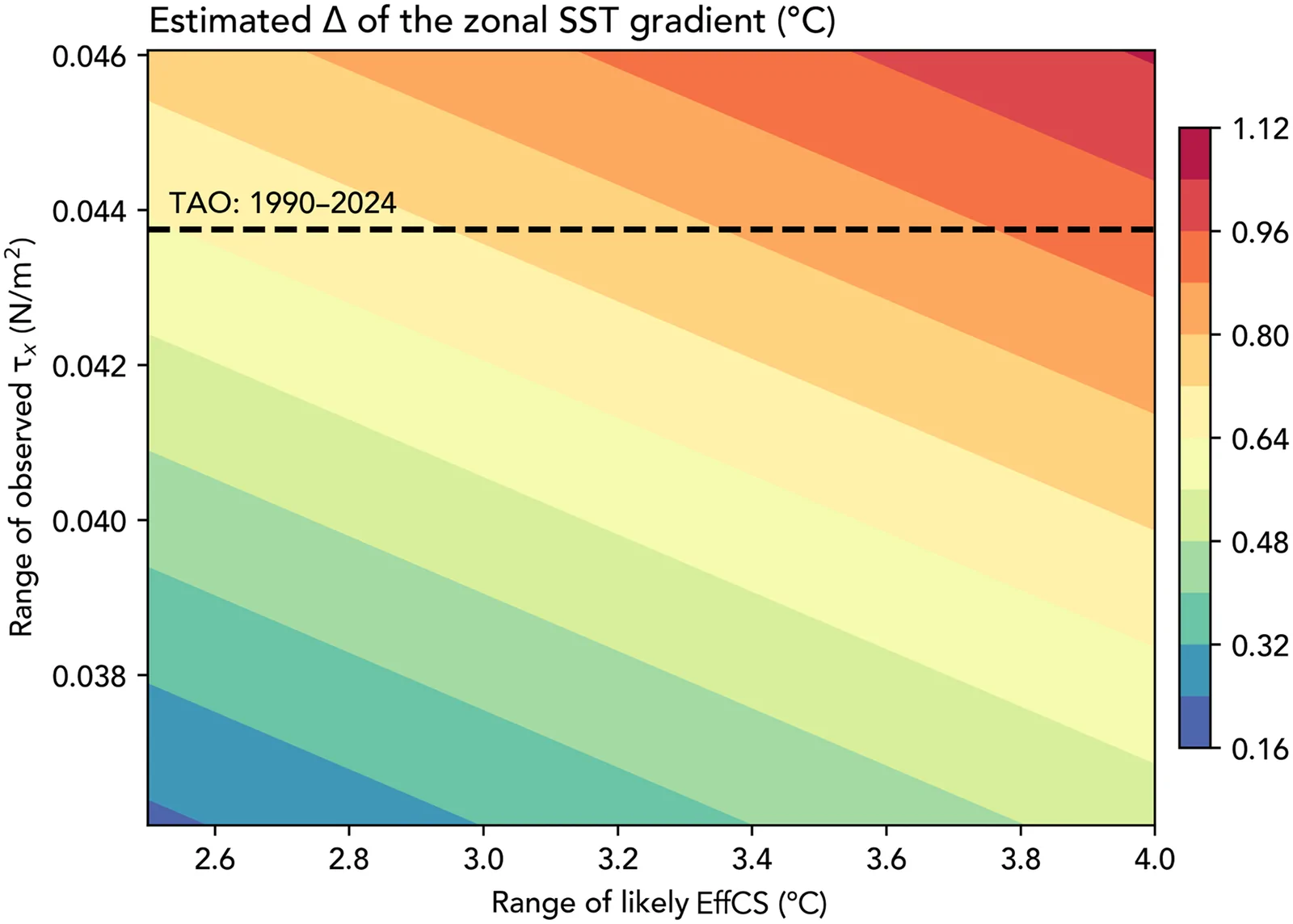
Estimated future changes in the zonal SST gradient. These values are predicted using the multiple regression of Fig. 2C from the range of likely EffCS and the range of the easterly trade wind strength from multiple reanalysis datasets (Materials and Methods). The horizontal dashed line indicates the climatology from the TAO observational array in the Tropical Pacific (150° to 95°W and 5°S to 5°N) as an example of direct measurements. Note that the strength of the trade winds estimated from reanalysis for 1900 to 1999 is 0.043 N/m^2^, close to the TAO array climatology. The multiple linear regression model describes the empirical relationship in (C), and was fit using CMIP6 data under SSP5-8.5, which is described in more detail in Materials and Methods.

Our numerical experiments with the mean-state modified CESM2 confirm the crucial role of background trade winds in the future warming patterns as seen across both CMIP6 models. The physical reasoning is straightforward: A model with a stronger mean Walker Circulation that maintains a large zonal SST gradient has more potential to experience the positive feedbacks associated with EEP warming. This includes the decrease in lower-tropospheric stability and low clouds in response to rising SSTs. For example, in our experiments during the first decades after CO_2_ quadrupling, net surface heat flux into the EEP is greatest in Strong Trades simulation, primarily due to enhanced shortwave input. Subsequently, the thermocline component of the Bjerknes feedback is ∼58% larger in the Strong Trades simulation than in the Weak Trades simulation, indicating a stronger feedback loop through which the east-west SST gradient can more efficiently erode.

Our work highlights competing theories between the changing tropical Pacific SST patterns and EffCS: Does EffCS depend on the future SST gradient change, or vice versa? We test this directly by examining EffCS across our three CESM2 experiments (fig. S10). The EffCS estimated via the method of Gregory *et al.* (*45*) remained largely unchanged across these experiments (Strong: 6.46°C, Moderate: 6.49°C, Weak: 6.06°C; fig. S10), with the modest 0.4°C reduction between the Strong and Weak Trades simulations possibly reflecting internal variability (*46*, *47*). This relative independence of EffCS from the simulated changes in the SST gradient is notable, considering the substantial literature linking tropical Pacific SST patterns to radiative feedbacks through low-cloud processes (*12*, *14*). One explanation that reconciles this result with the pattern effect literature is that EffCS depends more on warming over the western Pacific warm pool rather than the eastern Pacific (*48*, *49*), and our gradient changes are driven largely by eastern Pacific warming. Alternatively, the contribution of SST gradient change to EffCS is weaker than the pattern effect literature implies, or weaker in CESM2 specifically, and may be further obscured by compensating feedbacks, internal variability, or model biases.

We emphasize that accurate simulation of Pacific climatology and robust long-term observations of the tropical Pacific are essential not only for capturing ENSO dynamics but also for improving long-term climate projections under anthropogenic forcing and reducing their overall uncertainty (*17*, *21*). Other work has shown that applying flux adjustments to both SST and wind stress in CESM2 produces a more La Niña–like response to radiative forcing, consistent with our finding that mean-state trade wind strength modulates the magnitude of the forced SST gradient change (*50*). Therefore, historical biases associated with overly strong trade winds in CMIP6 models may partly explain much of the diversity in their projected tropical Pacific warming patterns and the discrepancies between model simulations and the recently observed decadal cooling trends in the EEP (*28*).

## MATERIALS AND METHODS

### CMIP6, CESM2 large ensemble, and reanalysis data

We make use of 25 CMIP6 models over a range of forcing scenarios: the piControl, Historical, SSP5-8.5, and the abrupt four-times CO_2_ experiments (*51*). Our multimodel ensemble means were calculated to equally weight each model, regardless of the number of ensemble members. All models containing SST and wind-stress data for the piControl, Historical, SSP5-8.5, and Abrupt 4 × CO_2_ scenarios on the Pangeo CMIP6 cloud archive were included, limiting the total number of suitable models to 25. EffCS values for each model were obtained from the results of (*12*). The zonal SST gradient is calculated as the difference between the EEP (5°S to 5°N and 140° to 80°W) and the western Pacific warm pool region (5°S to 5°N and 80° to 140°E), and the strength of the trade winds is calculated in each experiment as an average of 5°S to 5°N and 150° to 100°W.

All 100 members from the CESM2 large ensemble are used to test the relationships between the trade wind strength, temperature sensitivity to a CO_2_ increase, and the projected change in the zonal SST gradient (fig. S3). The CESM2 large ensemble does not include the 4 × CO_2_ scenario, so we estimate the unique sensitivity of each ensemble member to a CO_2_ increase as the change in global mean temperature from the Historical (1900 to 1999) to the SSP3-7.0 scenario (2050 to 2099).

For comparison of the model data with the observed period, we use multiple reanalysis datasets that cover different temporal ranges in the recent past and provide zonal wind-stress data. This includes ERA5 (1979 to 2020), ORAS5 (1958 to 2020), and the NOAA 20th Century Reanalysis version 3 (1900 to 1999) (*52*–*54*). An observational range of zonal-wind stress in the tropical Pacific (5°S to 5°N and 150°W to 100°W) is generated using bootstrap resampling of the monthly wind-stress to generate 1000 means per dataset. From these distributions, the 95% confidence intervals are extracted from each dataset. We proceed by using the lowest (from ERA5, 0.036 N/m^2^) and highest (from NOAA, 0.047 N/m^2^) confidence intervals across these datasets as estimates of the likely range of the observed values. For comparison with direct observations, we also use data from the TAO observational array in the tropical Pacific (*55*). We average zonal wind stress from the stations spanning 150°W to 95°W and 5°S to 5°N (15 total stations) from 1990 through 2024. The estimated trade wind strength from TAO, 0.044 N/m^2^, agrees well with the estimated range from reanalysis (Fig. 3).

### Modified mean-wind experiments using CESM2

We conduct targeted perturbation experiments using the Community Earth System Model version 2.1.3 (CESM2) (*36*) to explore how the mean state of the tropical Pacific influences the climate response to greenhouse gas forcing. Specifically, we apply reductions in momentum flux from the atmosphere to the ocean, effectively weakening the Pacific trade wind stress before the imposition of radiative forcing. This experimental setup enables us to isolate the influence of mean Pacific conditions within the same model while keeping climate sensitivity nearly constant.

In CESM2, the default simulation features a notably strong PWC and associated east-west SST gradient. We treat this control configuration as the Strong Trades state and make use of its publicly available piControl and abrupt-4×CO_2_ simulations from CMIP6. To create weaker trade wind configurations, we run two additional simulations with zonal wind stress reduced by about 10% and 20% in the central-eastern tropical Pacific (Fig. 4). These Moderate Trades and Weak Trades experiments are initiated from the CESM2 piControl run, wherein a zonal wind-stress perturbation is applied at each integration time step in the model’s coupler, which translates the atmospheric fluxes to the ocean and vice versa. The perturbation is applied using a westerly two-dimensional Gaussian perturbation centered in the central-eastern Pacific (see fig. S4). The perturbation is only applied at the point in which CESM2 translates the atmospheric momentum fluxes to the ocean surface.

The applied perturbation is a westerly two-dimensional Gaussian anomaly centered in the central-eastern Pacific (Eq. 1 and fig. S4).

$$\tau^{\prime }\left(x,y\right)=A\text{exp}\left\{-\left[\frac{{\left(x-\mu_{x}\right)}^{2}}{2\sigma_{x}^{2}}+\frac{{\left(y-\mu_{y}\right)}^{2}}{2\sigma_{y}^{2}}\right]\right\}$$ (1)

In this equation, *A* is the magnitude of the imposed zonal wind-stress perturbation, *x* and *y* are longitudinal and latitudinal coordinates, and $\mu_{x}$ and $\mu_{y}$ represent the coordinates of the center of the perturbation (120°W and 0°N). Last, $\sigma_{x}$ and $\sigma_{y}$ describe the standard length and width of the shape of the perturbation (set to 18°W/E and 6°N/S). For the magnitude A, we use 0.0145 N/m^2^ for the Moderate Trades case and 0.029 N/m^2^ for the Weak Trades case. These amplitudes allow us to reduce zonal wind stress by about 10 and 20%, respectively.

This approach offers two key advantages. First, because we do not impose direct surface heat fluxes changes, the radiative forcing associated with CO_2_ quadrupling is equivalent across all experiments, allowing for a clean comparison of the response. Second, the imposed reduction in wind stress yields a physically realistic mean-state SST pattern, characterized by reduced zonal gradients and an El Niño–like warming response. These modified mean states offer a controlled framework to investigate how the background structure of the tropical Pacific influences global feedbacks and temperature trajectories under enhanced greenhouse gas forcing.

Crucially, introducing this change does not lead to model drift or large deviations from the expected mean-state structure in the tropical Pacific (Fig. 3, A and B). In essence, the imposed reduction in the strength of the mean trade winds reduces the east-west SST gradient and decreases low-cloud cover in the EEP. The changes are amplified by the large-scale Bjerknes and low-cloud feedbacks operating across the equatorial Pacific. In addition, fig. S8 depicts climatological fields of SST, low-cloud concentrations, and $\tau_{x}$ from the control climates for all three experiments. The global mean surface temperature in these modified mean-state climates remains similar across all cases: 13.8°C for the Strong Trades, 14.1°C for the Moderate Trades, and 14.0°C for the Weak Trades, with differences comparable to those expected from internal variability.

### Estimating the Bjerknes feedback

To estimate the Bjerknes feedback, we focus on its largest component—the thermocline feedback. We follow an approach commonly used in studies of ENSO, involving the computation of the Bjerknes stability index (*39*). Here, however, we apply this method to longer timescales. This approach includes computing three coupling coefficients between different dynamical components of the tropical ocean-atmosphere system. The first, $\mu_{a}$, quantifies the atmospheric coupling: the sensitivity of equatorial central Pacific zonal wind stress anomalies $\left[\tau_{x}\right]$ to EEP SST anomalies $T_{E}$ (*2*). The second, $\beta_{h}$, captures the ocean dynamical coupling: the thermocline tilt response to wind forcing (*3*), where $h_{e}$ and $h_{w}$ are the eastern and western equatorial Pacific thermocline depth anomalies. The third, $a_{h}$, represents the subsurface-to-surface thermal coupling: the sensitivity of subsurface temperature anomalies ($T_{\text{sub}}$) to local thermocline depth variations (*4*). Each coefficient is estimated by linear regression of 5-year running mean filtered monthly anomalies, with statistical significance assessed using an effective sample size that accounts for the serial correlation introduced by the smoothing (fig. S9).

The averaging regions for wind stress and SST follow (*39*): $\left[\tau_{x}\right]$ is averaged over the central equatorial Pacific (5°S to 5°N, 150°E to 130°W) and $T_{E}$ over the Niño-3 region (5°S to 5°N, 150°W to 90°W). The eastern and western thermocline depths $h_{e}$ and $h_{w}$, and the subsurface temperature $T_{\text{sub}}$, are averaged over the eastern (5°S to 5°N, 140°W to 80°W) and western (5°S to 5°N, 120°E to 180°W) equatorial Pacific. $T_{\text{sub}}$ is evaluated at a fixed depth of 85 m, consistent with the mean thermocline depth across experiments [74 to 84 m; cf. the 75 m used in (*39*)]; a fixed reference level is retained for $a_{h}$ so that the regression isolates the subsurface temperature response to thermocline displacement rather than to a comoving reference surface.

$$\left[\tau_{x}\right]=\mu_{a}\;T_{E}\left(\text{atmospheric coupling}\right)$$ (2)

$$h_{e}-h_{w}=\beta_{h}\;\left[\tau_{x}\right]\left(\text{ocean dynamical coupling}\right)$$ (3)

$$T_{\text{sub}}=a_{h}\;h_{e}\left(\text{subsurface temperature coupling}\right)$$ (4)

The product $\mu_{a}\cdot \beta_{h}\cdot a_{h}$ provides a scalar measure of the strength of the Bjerknes thermocline feedback loop: how efficiently an SST perturbation in the EEP drives a wind stress response, which reorganizes the equatorial thermocline, which in turn modifies subsurface temperatures that feed back onto SST through vertical advection. A larger absolute value of this product indicates a stronger positive feedback. Here, we compare the Bjerknes feedback across three control experiments with differing mean trade wind strengths to assess how the low-frequency background circulation modulates the coupled instability that governs the east-west temperature contrast.

We additionally evaluate the net upwelling contribution to the growth rate. Following equation 6.15 of (*39*), and excluding the horizontal mean-current damping and the zonal, meridional, and vertical advective feedback terms, this net contribution is the differenceof the thermocline feedback and the mean-stratification damping

$$R_{\text{TH}}=\underset{\text{TH}}{\underbrace{\mu_{a}\beta_{h}a_{h}\;\gamma \;\frac{\bar{w}}{H_{m}}}}-\underset{\text{DD}\;\left(\text{vertical}\right)}{\underbrace{\gamma \;\frac{\bar{w}}{H_{m}}}}$$ (5)

where γ=0.75 is the vertical entrainment efficiency (*39*) and $M\left(\bar{w}\right)=1$ in the upwelling-favorable eastern Pacific. We take $\bar{w}$ as the time-mean vertical velocity at the mean eastern-Pacific thermocline depth and adopt this same depth (the 20°C isotherm depth) as the vertical scale $H_{m}$, evaluated separately for each experiment to reflect its distinct mean state.

We compare the magnitude of the Bjerknes feedback, *R*_TH_, across three modified wind-stress experiments using the mean state from the 100 years preceding the application of the 4×CO_2_ forcing. We find that *R*_TH_ increases monotonically with mean trade wind strength (Fig. 5G), implying that initial SST anomalies in the EEP induced by radiative forcing will experience greater amplification in the Strong Trades experiment.

There are several caveats to keep in mind. The Bjerknes Stability Index was originally developed for ENSO and is based on the recharge oscillator framework, which may need to be modified for the decadal and longer timescales relevant to this study. Nevertheless, we tested the method by applying a 10-year rolling mean to the data instead of a 5-year rolling mean. The results remained unchanged, although the number of degrees of freedom decreased because we considered 100-year intervals.

## Supplementary Materials

### This PDF file includes:

Table S1 Figs. S1 to S10 References
