# Supplementary material for: Background trade winds and climate sensitivity control the eastern equatorial Pacific warming pattern
Source: Sci Adv. 2026 Sep 30;12(40):eaec5746. doi: 10.1126/sciadv.aec5746 (PMC13626047; doi:10.1126/sciadv.aec5746)
Supplement: Supplementary file 1 [file sciadv.aec5746_sm.pdf]

Science Advances
AAAS

Supplementary Materials for

Background trade winds and climate sensitivity control the eastern equatorial Pacific warming pattern

Jacob Stuivenvolt-Allen and Alexey V. Fedorov

Corresponding author: Jacob Stuivenvolt-Allen, jsallen@ucar.edu

Sci. Adv. **12**, eaec5746 (2026)
DOI: 10.1126/sciadv.aec5746

This PDF file includes:

Table S1
Figs. S1 to S10
References

Table S1: CMIP6 global climate models used in this study, their developing institutions, and key model description references.

**Model**	**Institution**	**Reference**
ACCESS-CM2	CSIRO–ARCCSS	(*57*)
ACCESS-ESM1-5	CSIRO	(*58*)
BCC-CSM2-MR	BCC	(*59*)
BCC-ESM1	BCC	(*60*)
CESM2	NCAR	(*36*)
CESM2-WACCM	NCAR	(*36*)
CNRM-CM6-1	CNRM–CERFACS	(*76*)
CNRM-ESM2-1	CNRM–CERFACS	(*61*)
CanESM5	CCCma	(*62*)
E3SM-1-0	E3SM Project	(*63*)
EC-Earth3	EC-Earth Consortium	(*64*)
EC-Earth3-Veg	EC-Earth Consortium	(*64*)
GFDL-CM4	NOAA-GFDL	(*65*)
GFDL-ESM4	NOAA-GFDL	(*66*)
GISS-E2-1-G	NASA-GISS	(*67*)
GISS-E2-1-H	NASA-GISS	(*67*)
HadGEM3-GC31-LL	MOHC	(*68*)
INM-CM4-8	INM	(*69*)
INM-CM5-0	INM	(*70*)
IPSL-CM6A-LR	IPSL	(*77*)
MIROC-ES2L	MIROC	(*71*)
MIROC6	MIROC	(*72*)
MRI-ESM2-0	MRI	(*78*)
NESM3	NUIST	(*73*)
SAM0-UNICON	SNU	(*74*)
UKESM1-0-LL	MOHC–NERC	(*75*)

Supplementary figures

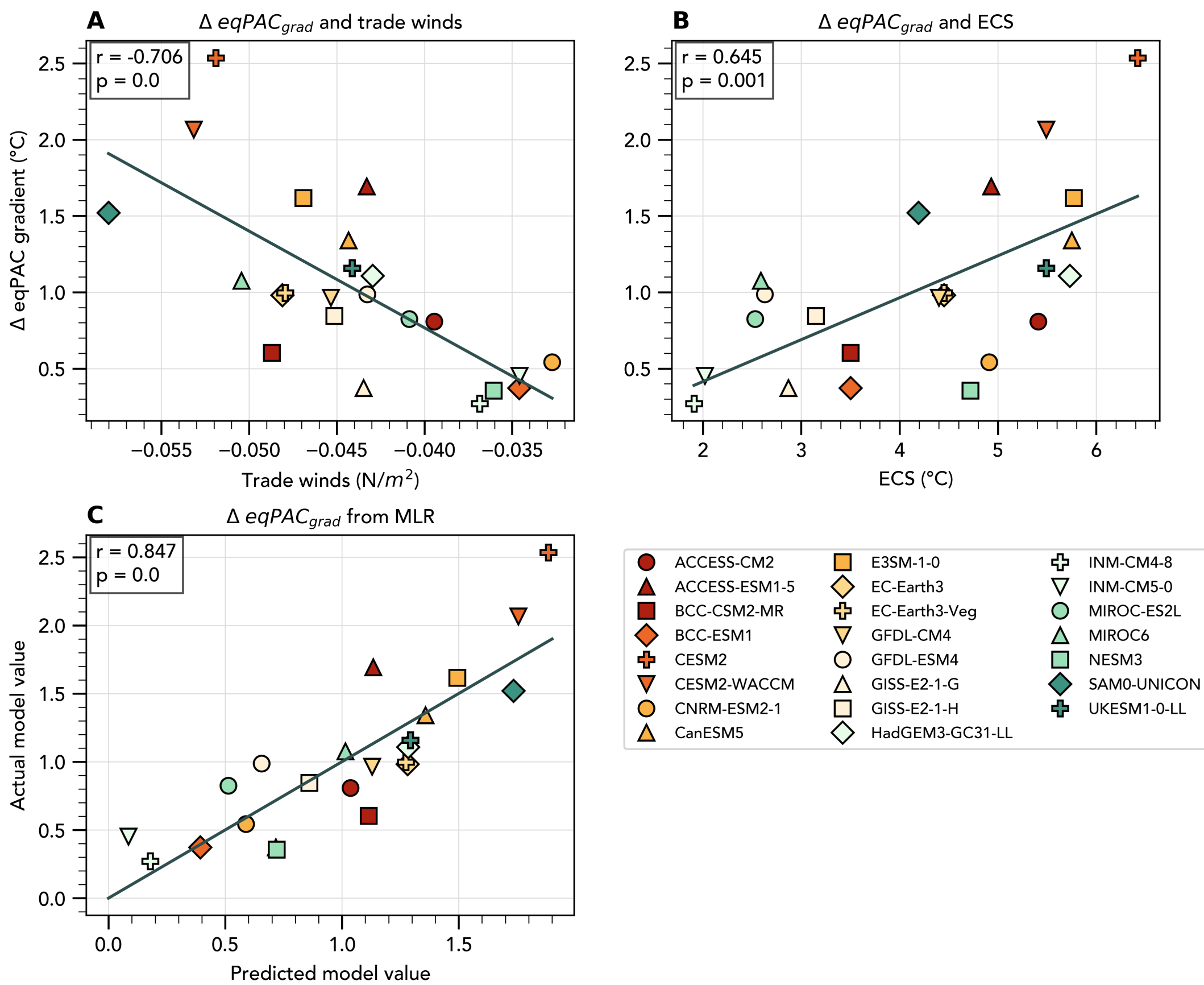

Figure S1: **Projected changes in the equatorial Pacific zonal SST gradient under 4xCO2 as a function of background trade-wind strength and EffCS.** (A) Scatter plot of the projected change in the SST gradient after CO2 quadrupling versus background trade-wind strength in the preindustrial control experiments for each CMIP6 model. (B) Projected change in the equatorial SST gradient versus each model's EffCS. (C) EffCS and the trade-wind strength combined as predictors of the future change in the equatorial SST gradient using multiple linear regression. Correlation coefficients from the linear fits are provided in the top-left of each subplot.

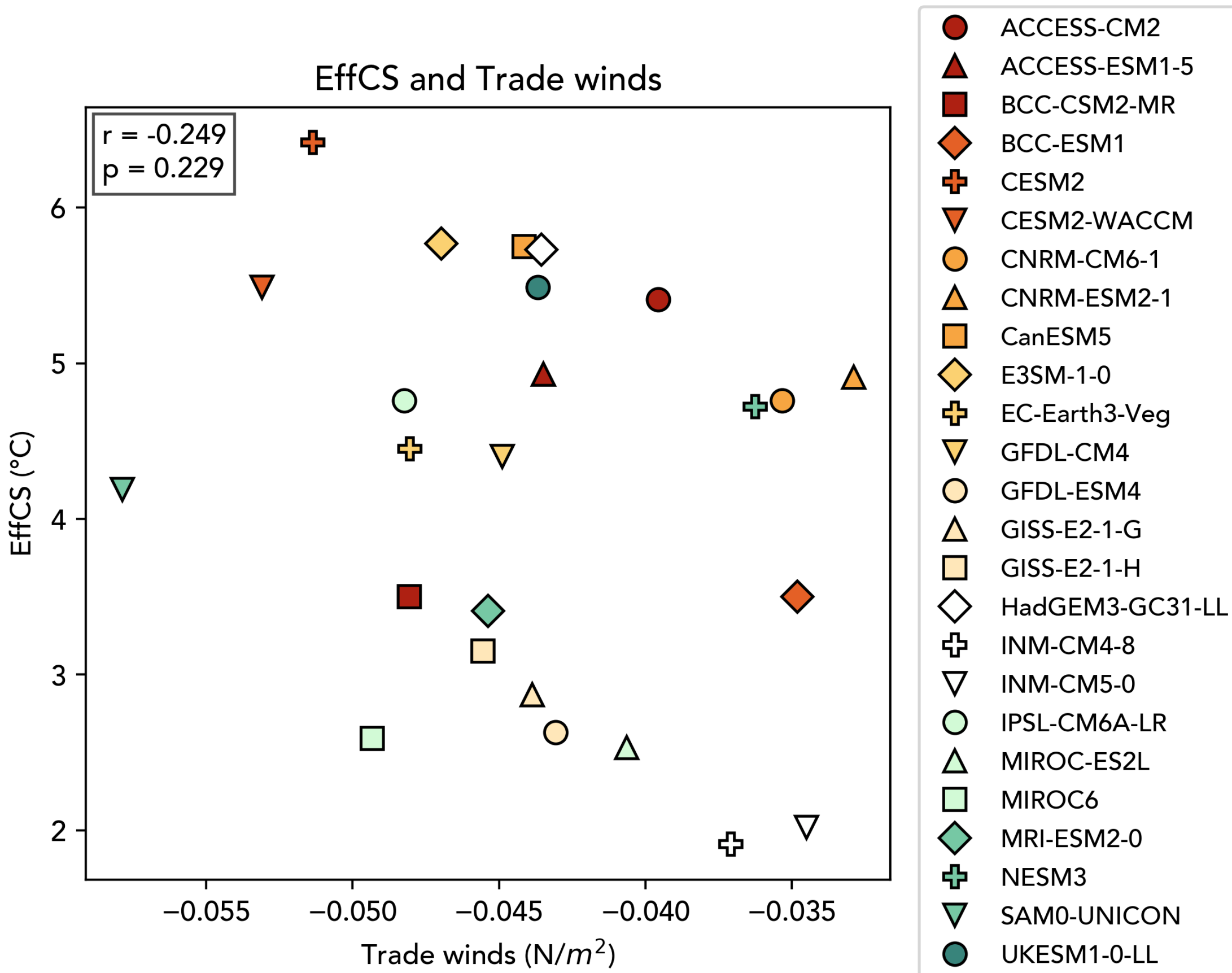

Figure S2: **Relationship between EffCS and the trade-wind strength in CMIP6 models.** Scatter plot of a model's EffCS as estimated from an abrupt 4xCO_2 experiment, along with its mean trade-wind strength from the Historical scenario (1900-1999). The r-value and p-value from the correlation are included in the top-left of the plot.

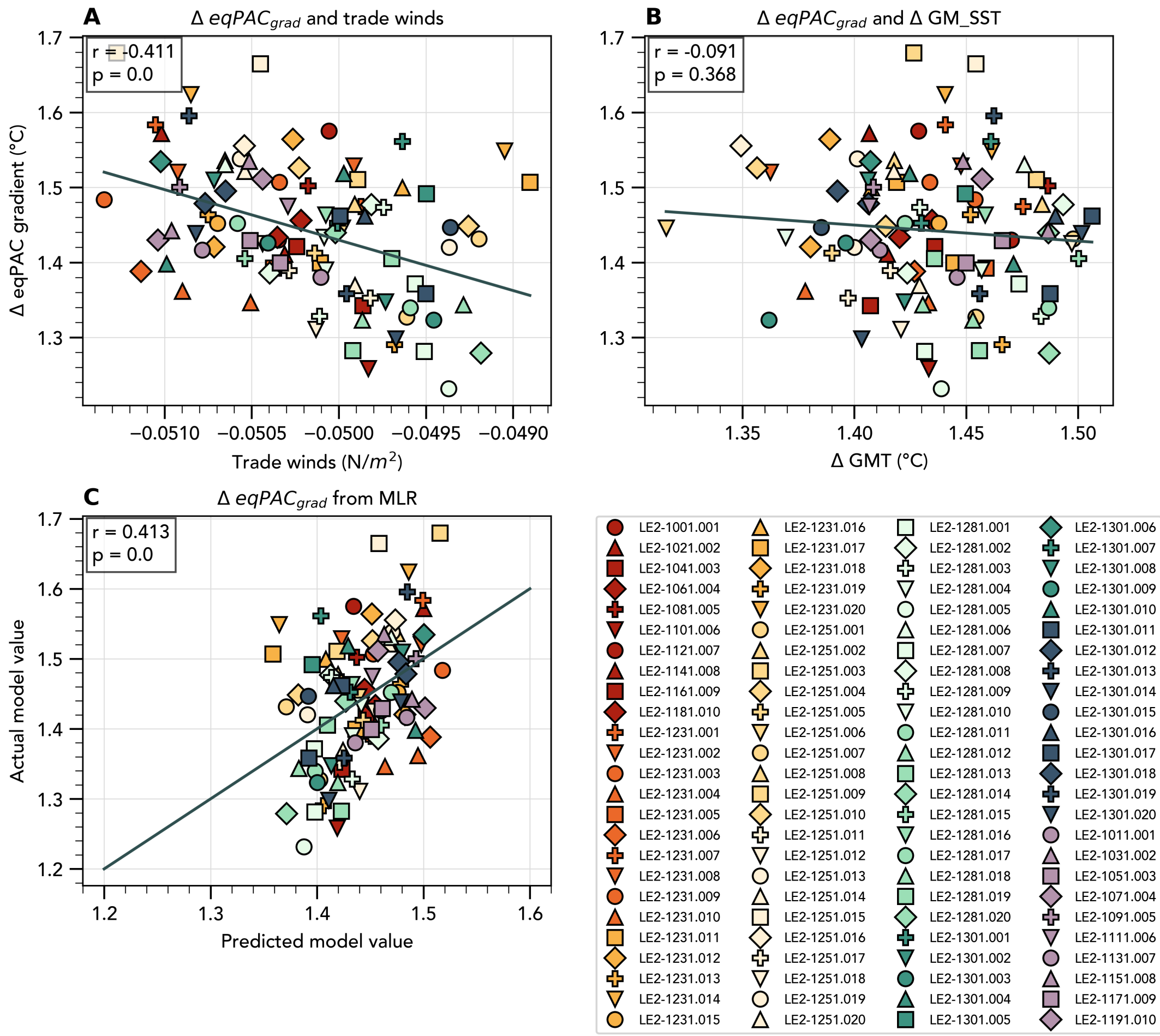

Figure S3: **Projected changes in the equatorial Pacific zonal SST gradient in a large ensemble.** Scatter plot of the projected change in the SST gradient under the SSP3-7.0 scenario versus background trade-wind strength in the Historical scenario (1900-1999) for each member of the CESM2 Large Ensemble. (B) Projected change in the equatorial SST gradient versus each ensemble member's global mean surface temperature change. No significant correlation is found between the two variables, because most of the differences reflect internal variability. (C) The global mean temperature change and mean trade-wind strength combined as predictors of the future change in the equatorial SST gradient using multiple linear regression. Correlation coefficients from the linear fits are provided in the top-left of each subplot.

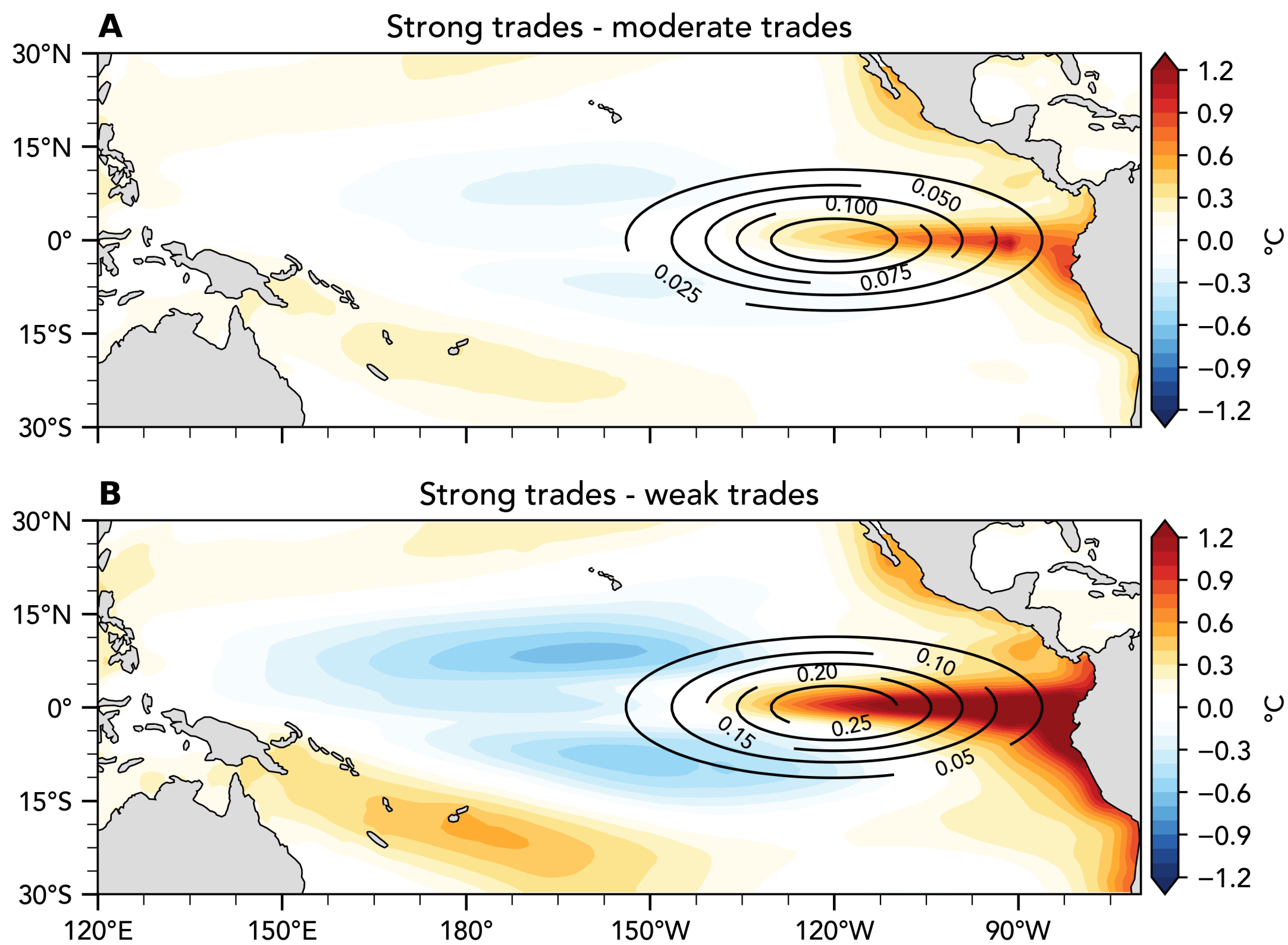

Figure S4: **Imposed wind-stress perturbations and the SST response in the modified mean-state experiments.** (A) The imposed westerly wind stress perturbation (contours, in dyn/m^2) and the resulting SST anomaly (color shading, °C) in the *Moderate Trades* experiment. The SST anomaly is computed as the difference between the *Moderate Trades* and the *Strong Trades* experiments over the first 100 years of the simulations (before the 4xCO2 forcing was applied). (B) As in panel (A), but for the *Weak Trades* experiment, which uses a larger wind-stress perturbation. Note the stationary Kelvin and Rossby wave patterns induced by the wind-stress perturbations

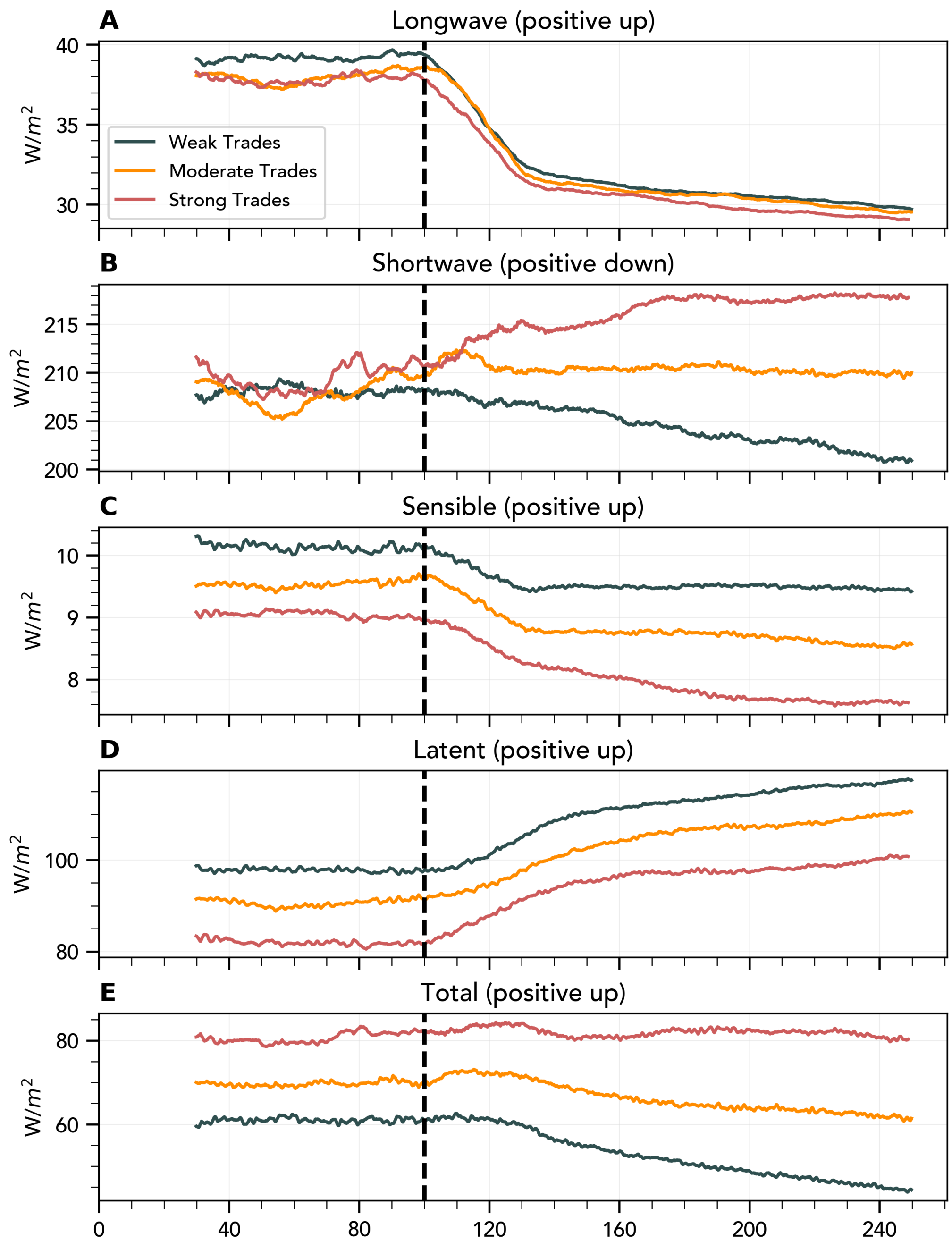

Figure S5: **Evolution of surface heat fluxes averaged over the eastern equatorial Pacific in the modified wind-stress experiments.** (A)-(E) Surface longwave, shortwave, sensible, latent, and total heat flux, respectively. Red, orange, and gray lines correspond to the *Strong*, *Moderate*, and *Weak Trades* experiments, respectively. The fluxes are averaged over 5°S-5°N and 100°-80°W, with a 30-year rolling mean applied to reduce the effects of internal variability. The total surface heat flux is computed as the shortwave flux (positive downward into the ocean) minus the sum of the longwave, sensible, and latent heat fluxes. The dashed line indicates the time at which CO2 was quadrupled.

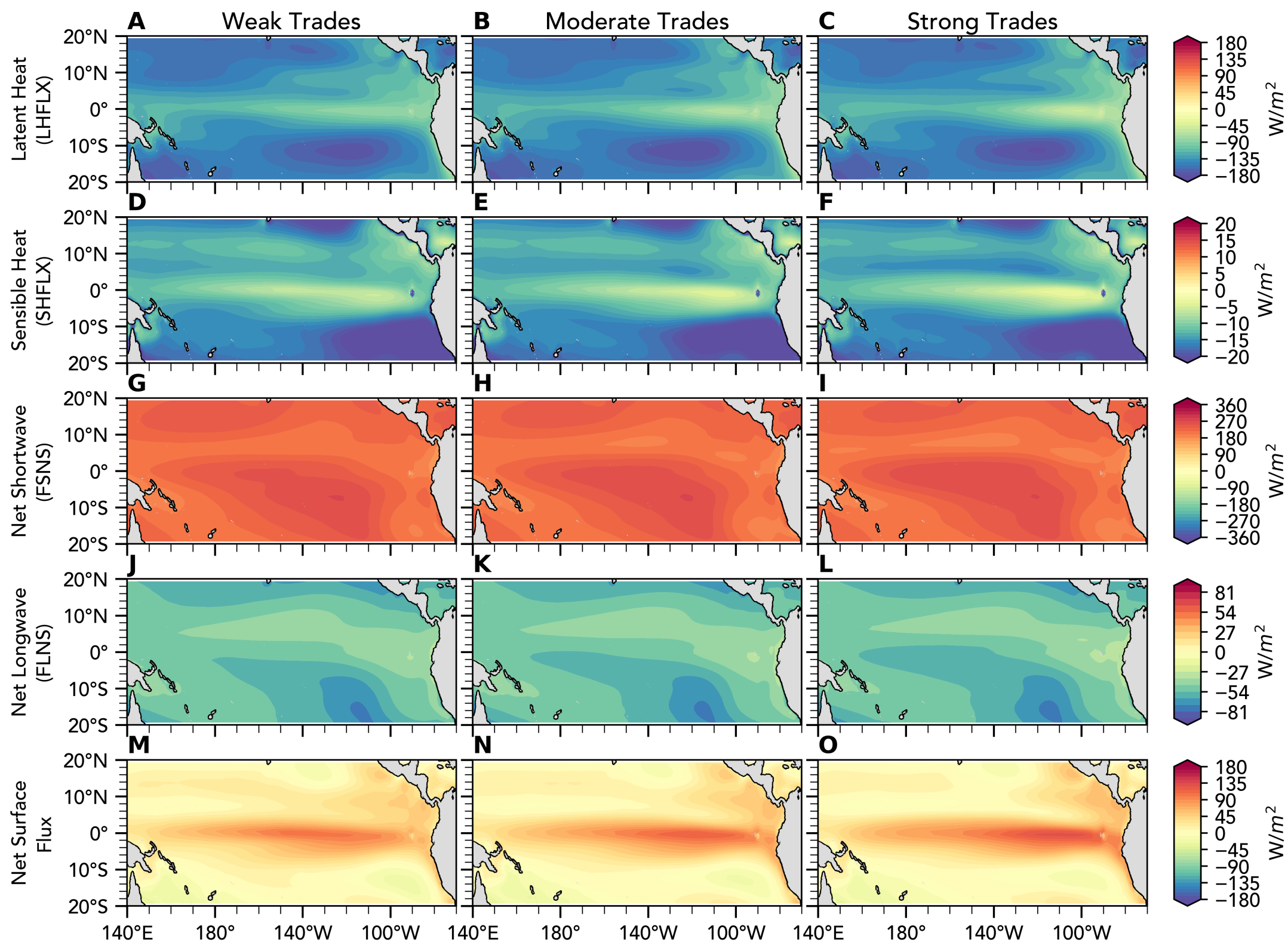

Figure S6: **Climatological surface heat fluxes in the tropical Pacific in the modified wind-stress experiments** The fluxes are averaged for the first 100 years, preceding CO_2 quadrupling. The left, middle, and right columns correspond to the *Strong*, *Moderate*, and *Weak Trades* experiments, respectively.

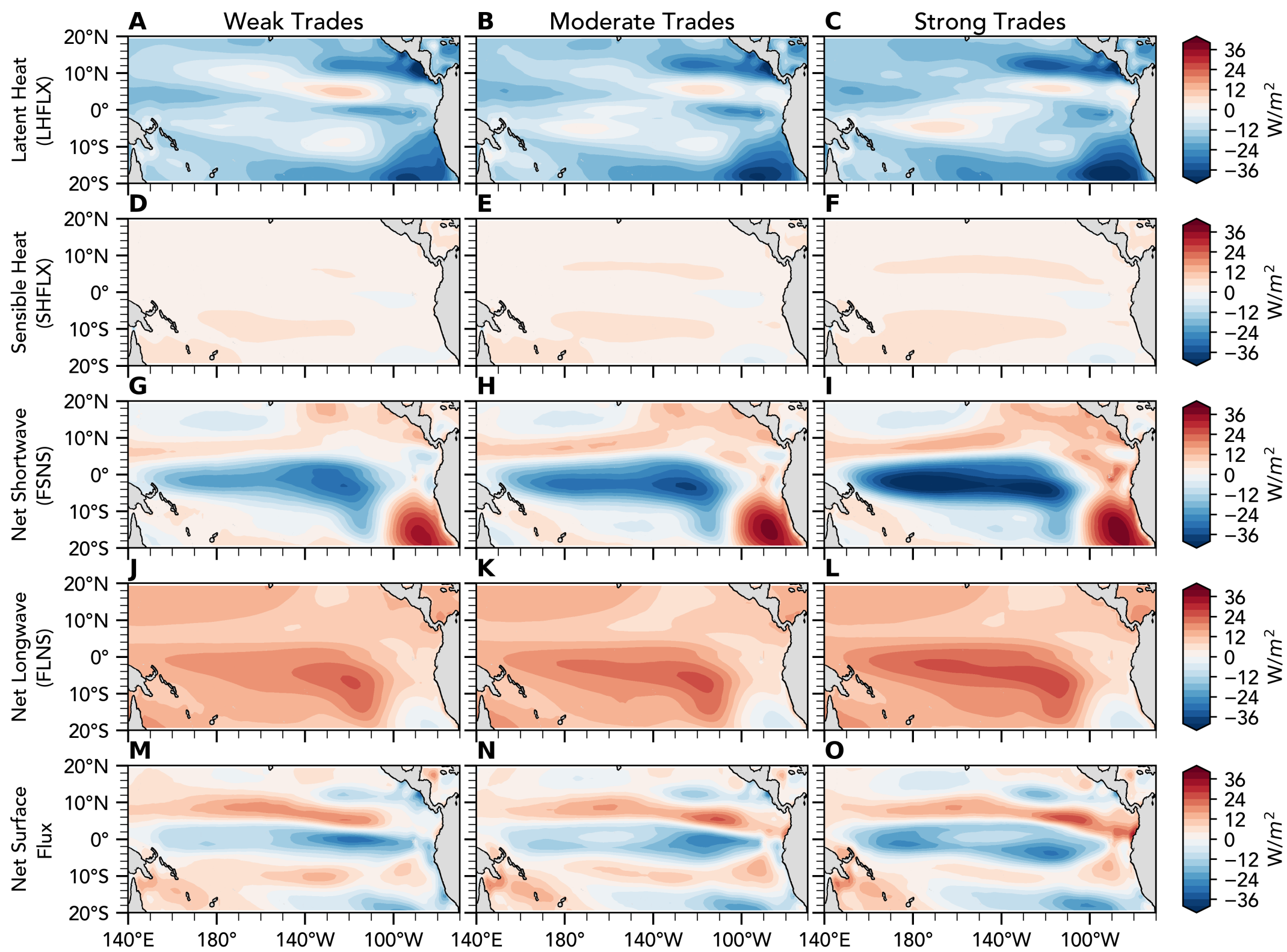

Figure S7: **Difference in surface heat fluxes after quadrupling CO_2.** As in Fig. S6, but for anomalies in surface heat fluxes after CO2 quadrupling. Each panel shows anomalies averaged over the last 100 years of the experiments relative to the first 100 years (before the 4xCO2 forcing was applied).

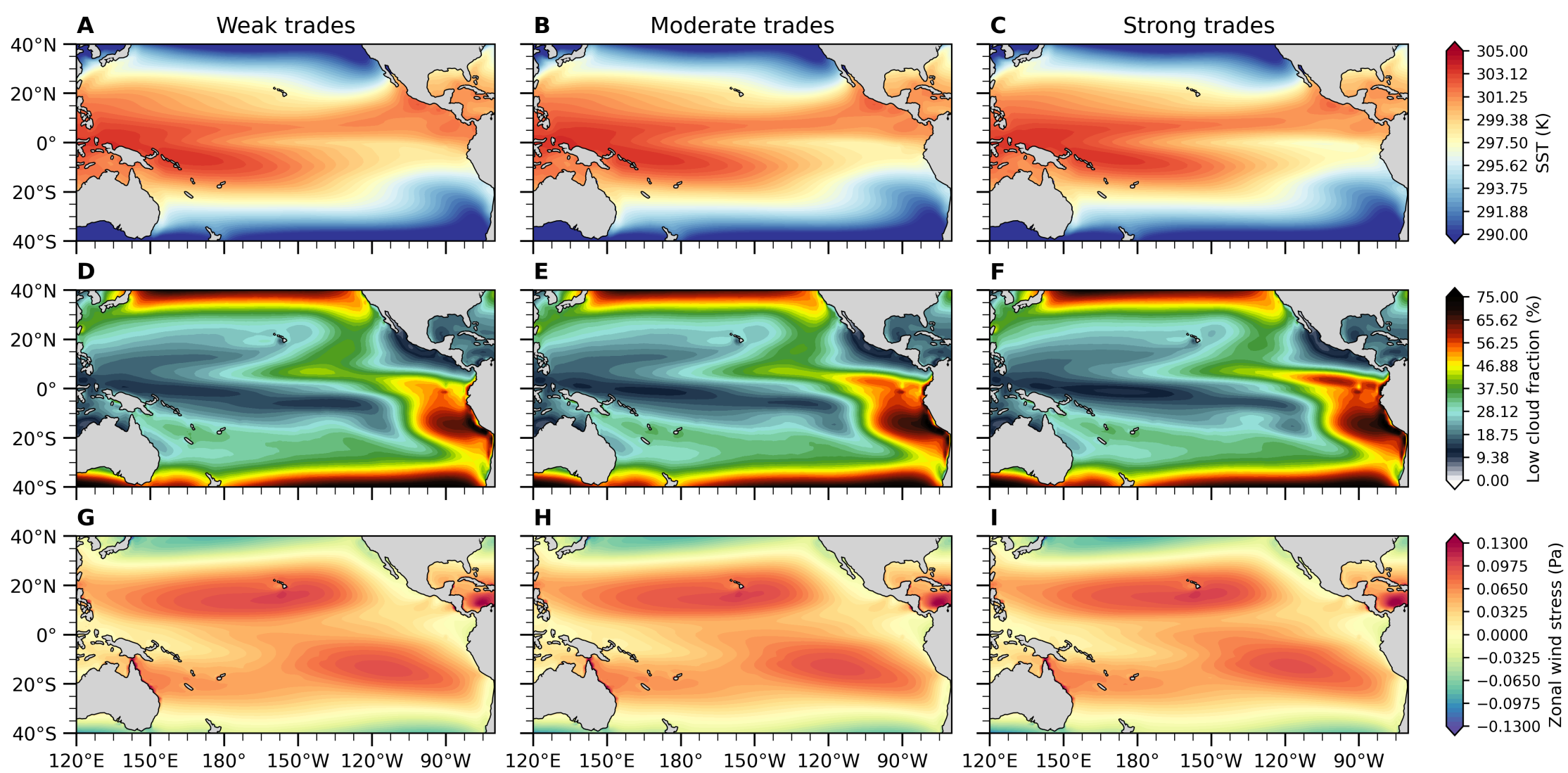

Figure S8: **The tropical Pacific mean state in the modified wind-stress experiments.** (A-C) Climatological SST, (D-F) low-cloud fraction, and (G-I) zonal wind stress. The left, middle, and right columns correspond to the *Strong*, *Moderate*, and *Weak Trades* experiments, respectively.

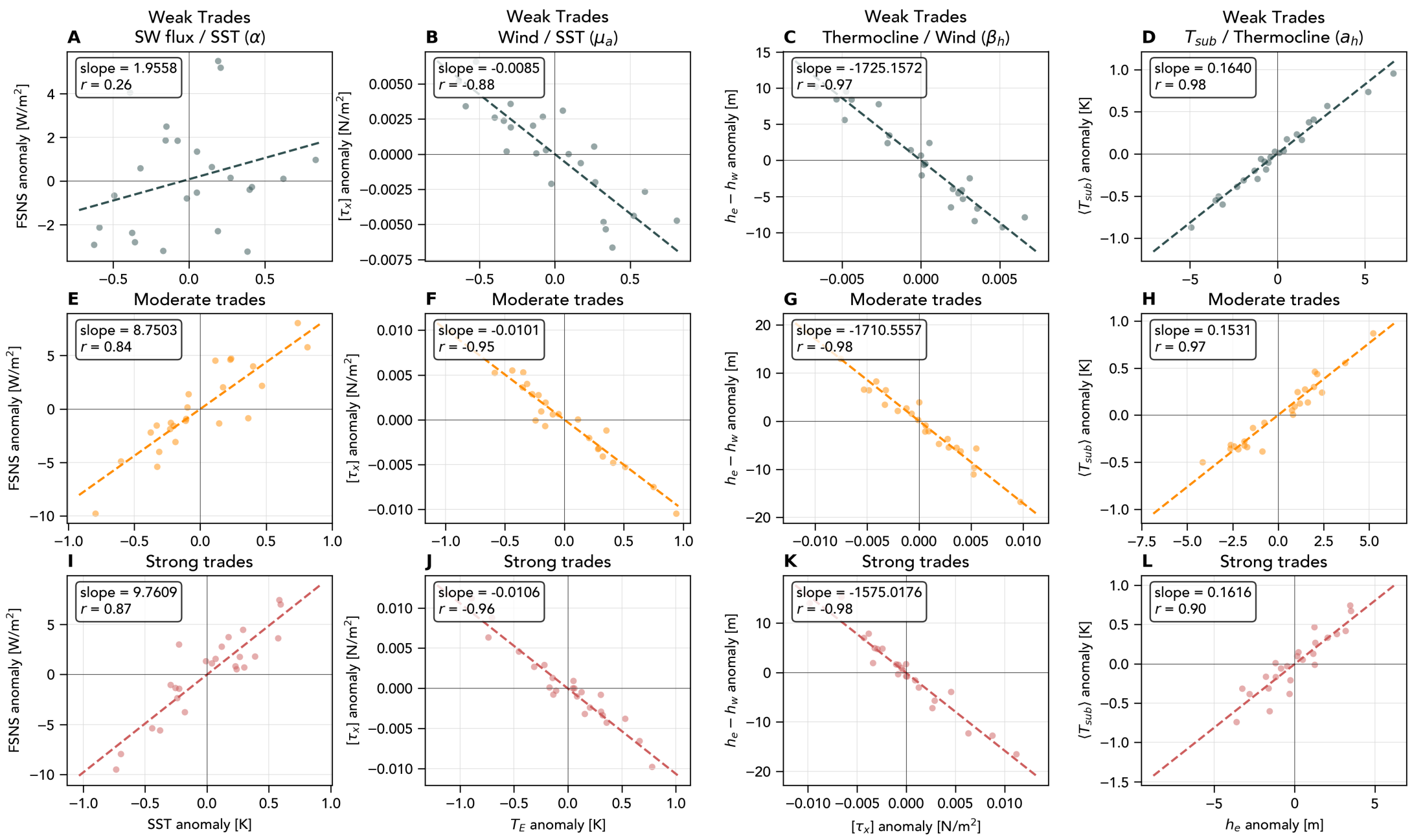

Figure S9: **Scatter plots used to compute four ocean-atmosphere coupling coefficients in the modified wind-stress experiments** The top, middle, and bottom rows correspond to the *Strong*, *Moderate*, and *Weak Trades* experiments, respectively. (A),(E),(I) Shortwave flux–SST coupling coefficient (α), with net surface shortwave radiation anomalies regressed against SST anomalies over the eastern equatorial Pacific. (B),(F),(J) Wind stress–SST coupling (μ_a), with central Pacific zonal wind stress anomalies regressed against Niño-3 SST anomalies. (C),(G),(K) Thermocline–wind stress coupling (β_h), with the east-west thermocline depth gradient anomaly regressed against central Pacific wind stress anomalies. (D),(H),(L) Subsurface temperature–thermocline coupling (a_h), with subsurface temperature anomalies regressed against eastern equatorial thermocline depth anomalies. A 5-year rolling mean has been applied to each timeseries to separate lower-frequency variations from interannual variability. Dashed lines show linear regressions, with slope and correlation coefficient (r) annotated using 20 effective degrees of freedom.

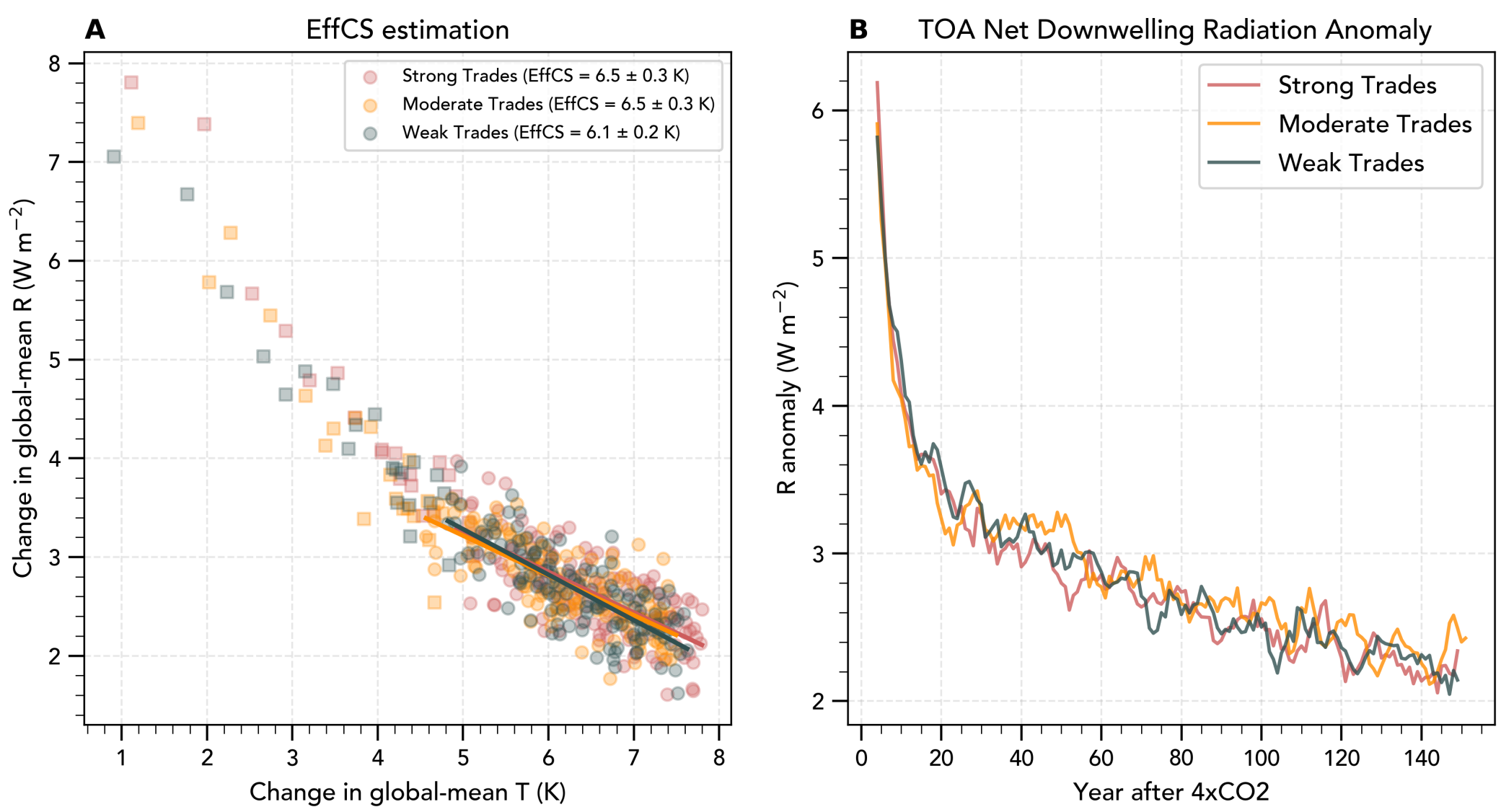

Figure S10: **Gregory regression plots used to estimate EffCS in the modified wind-stress experiments.** (A) Changes in the global mean net top-of-atmosphere radiation imbalance versus changes in global mean surface temperature. The plot shows annual mean anomalies relative to the 100-year interval preceding CO2 quadrupling (Fig. 3c). (B) Net top-of-atmosphere radiation imbalance after CO2 quadrupling. Following (*45*), EffCS is computed as EffCS = -intercept/(2·slope). The slope is estimated using least squares. The standard error of EffCS is estimated via the delta method, which propagates the uncertainties in the slope and intercept, along with their covariance.

REFERENCES

1. S. Gopika, M. Lengaigne, I. Suresh, T. Izumo, S. Kwatra, S. Neetu, J. Vialard, Assessing CMIP models' ability to detect observed surface warming signals related to climate change. *J. Clim.* **37**, 6011–6027 (2024).

2. I. M. Held, B. J. Soden, Robust responses of the hydrological cycle to global warming. *J. Clim.* **19**, 5686–5699 (2006).

3. U. K. Heede, A. V. Fedorov, N. J. Burls, Time scales and mechanisms for the tropical Pacific response to global warming: A tug of war between the ocean thermostat and weaker Walker. *J. Clim.* **33**, 6101–6118 (2020).

4. J. Ying, M. Collins, W. Cai, A. Timmermann, P. Huang, D. Chen, K. Stein, Emergence of climate change in the tropical Pacific. *Nat. Clim. Change* **12**, 356–364 (2022).

5. V. Danielli, M. Lengaigne, K. Sadhvi, S. Gopika, J. Vialard, Drivers of CMIP tropical pacific warming pattern diversity. *Earth's Future* **13**, e2025EF005938 (2025).

6. W. Cai, B. Ng, G. Wang, A. Santoso, L. Wu, K. Yang, Increased ENSO sea surface temperature variability under four IPCC emission scenarios. *Nat. Clim. Change* **12**, 228–231 (2022).

7. W. Cai, B. Ng, T. Geng, Anthropogenic impacts on twentieth-century ENSO variability changes. *Nat. Rev. Earth Environ.* **4**, 407–418 (2023).

8. A. I. Williams, N. Jeevanjee, J. Bloch-Johnson, Circus tents, convective thresholds, and the non-linear climate response to tropical SSTs. *Geophys. Res. Lett.* **50**, e2022GL101499 (2023).

9. Y. Kosaka, S.-P. Xie, The tropical Pacific as a key pacemaker of the variable rates of global warming. *Nat. Geosci.* **9**, 669–673 (2016).

10. T. Andrews, J. M. Gregory, D. Paynter, L. G. Silvers, C. Zhou, T. Mauritsen, M. J. Webb, K. C. Armour, P. M. Forster, H. Titchner, Accounting for changing temperature patterns

increases historical estimates of climate sensitivity. *Geophys. Res. Lett.* **45**, 8490–8499 (2018).

11. B. Stevens, S. C. Sherwood, S. Bony, M. J. Webb, Prospects for narrowing bounds on Earth's equilibrium climate sensitivity. *Earth's Future* **4**, 512–522 (2016).

12. Y. Dong, K. C. Armour, M. D. Zelinka, C. Proistosescu, D. S. Battisti, C. Zhou, T. Andrews, Intermodel spread in the pattern effect and its contribution to climate sensitivity in CMIP5 and CMIP6 models. *J. Clim.* **33**, 7755–7775 (2020).

13. G. A. Meehl, C. A. Senior, V. Eyring, G. Flato, J. F. Lamarque, R. J. Stouffer, K. E. Taylor, M. Schlund, Context for interpreting equilibrium climate sensitivity and transient climate response from the CMIP6 Earth system models. *Sci. Adv.* **6**, eaba1981 (2020).

14. M. D. Zelinka, T. A. Myers, D. T. McCoy, S. Po-Chedley, P. M. Caldwell, P. Ceppi, S. A. Klein, K. E. Taylor, Causes of higher climate sensitivity in CMIP6 models. *Geophys. Res. Lett.* **47**, e2019GL085782 (2020).

15. J. Ying, M. Collins, R. Chadwick, S. Chen, X. Hu, T. Lian, S. Long, Causes of differences in the tropical pacific sst warming pattern projected by CMIP6 models. *Adv. Atmos. Sci.* **42**, 1077–1091 (2025).

16. T. Andrews, A. Bodas-Salcedo, J. M. Gregory, Y. Dong, K. C. Armour, D. Paynter, P. Lin, A. Modak, T. Mauritsen, J. N. S. Cole, B. Medeiros, J. J. Benedict, H. Douville, R. Roehrig, T. Koshiro, H. Kawai, T. Ogura, J. L. Dufresne, R. P. Allan, C. Liu, On the effect of historical SST patterns on radiative feedback. *J. Geophys. Res. Atmos.* **127**, e2022JD036675 (2022).

17. T. R. Knutson, S. Manabe, Time-mean response over the tropical Pacific to increased CO_2 in a coupled ocean-atmosphere model. *J. Clim.* **8**, 2181–2199 (1995).

18. T. M. Merlis, T. Schneider, Changes in zonal surface temperature gradients and Walker circulations in a wide range of climates. *J. Clim.* **24**, 4757–4768 (2011).

19. N. Burls, A. Fedorov, What controls the mean east–west sea surface temperature gradient in the equatorial Pacific: The role of cloud albedo. *J. Clim.* **27**, 2757–2778 (2014).

20. U. K. Heede, A. V. Fedorov, N. J. Burls, A stronger versus weaker Walker: Understanding model differences in fast and slow tropical Pacific responses to global warming. *Clim. Dyn.* **57**, 2505–2522 (2021).

21. A. Fedorov, N. Burls, K. Lawrence, L. Peterson, Tightly linked zonal and meridional sea surface temperature gradients over the past five million years. *Nat. Geosci.* **8**, 975–980 (2015).

22. Z. I. Espinosa, M. D. Zelinka, C. M. Bitz, K. C. Armour, The strength of coupling to the Southern Ocean modulates Tropical Eastern Pacific variability and forced response. *J. Clim.* **39**, e250226 (2026).

23. M. Fu, A. Fedorov, The role of Bjerknes and shortwave feedbacks in the tropical Pacific SST response to global warming. *Geophys. Res. Lett.* **50**, e2023GL105061 (2023).

24. S.-P. Xie, C. Deser, G. A. Vecchi, J. Ma, H. Teng, A. T. Wittenberg, Global warming pattern formation: Sea surface temperature and rainfall. *J. Clim.* **23**, 966–986 (2010).

25. G. A. Vecchi, B. J. Soden, A. T. Wittenberg, I. M. Held, A. Leetmaa, M. J. Harrison, Weakening of tropical Pacific atmospheric circulation due to anthropogenic forcing. *Nature* **441**, 73–76 (2006).

26. U. K. Heede, A. V. Fedorov, Eastern equatorial Pacific warming delayed by aerosols and thermostat response to CO_2 increase. *Nat. Clim. Change* **11**, 696–703 (2021).

27. A. C. Clement, R. Seager, M. A. Cane, S. E. Zebiak, An ocean dynamical thermostat. *J. Clim.* **9**, 2190–2196 (1996).

28. R. Seager, M. Cane, N. Henderson, D. E. Lee, R. Abernathey, H. Zhang, Strengthening tropical Pacific zonal sea surface temperature gradient consistent with rising greenhouse gases. *Nat. Clim. Change* **9**, 517–522 (2019).

29. U. K. Heede, A. V. Fedorov, Towards understanding the robust strengthening of ENSO and more frequent extreme El Niño events in CMIP6 global warming simulations. *Clim. Dyn.* **61**, 3047–3060 (2023).

30. M. Latif, T. Bayr, J. Kjellsson, J. F. Lübbecke, T. Martin, H. C. Nnamchi, W. Park, A. Savita, J. Sun, D. Dommenget, Strengthening atmospheric circulation and trade winds slowed tropical Pacific surface warming. *Commun. Earth Environ.* **4**, 249 (2023).

31. P. N. DiNezio, G. A. Vecchi, A. C. Clement, Detectability of changes in the Walker circulation in response to global warming. *J. Clim.* **26**, 4038–4048 (2013).

32. U. K. Heede, A. V. Fedorov, Colder eastern equatorial Pacific and stronger Walker circulation in the early 21st century: Separating the forced response to global warming from natural variability. *Geophys. Res. Lett.* **50**, e2022GL101020 (2023).

33. S. Lee, M. L'Heureux, A. T. Wittenberg, R. Seager, P. A. O'Gorman, N. C. Johnson, On the future zonal contrasts of equatorial Pacific climate: Perspectives from observations, simulations, and theories. *Npj Clim. Atmos. Sci.* **5**, 82 (2022).

34. S. M. Kang, Y. Shin, H. Kim, S.-P. Xie, S. Hu, Disentangling the mechanisms of equatorial Pacific climate change. *Sci. Adv.* **9**, eadf5059 (2023).

35. M. Rugenstein, J. Bloch-Johnson, J. Gregory, T. Andrews, T. Mauritsen, C. Li, T. L. Frölicher, D. Paynter, G. Danabasoglu, S. Yang, J. L. Dufresne, L. Cao, G. A. Schmidt, A. Abe-Ouchi, O. Geoffroy, R. Knutti, Equilibrium climate sensitivity estimated by equilibrating climate models. *Geophys. Res. Lett.* **47**, e2019GL083898 (2020).

36. G. Danabasoglu, J.-F. Lamarque, J. Bacmeister, D. A. Bailey, A. K. Du Vivier, J. Edwards, L. K. Emmons, J. Fasullo, R. Garcia, A. Gettelman, C. Hannay, M. M. Holland, W. G. Large, P. H. Lauritzen, D. M. Lawrence, J. T. M. Lenaerts, K. Lindsay, W. H. Lipscomb, M. J. Mills, R. Neale, K. W. Oleson, B. Otto-Bliesner, A. S. Phillips, W. Sacks, S. Tilmes, L. van Kampenhout, M. Vertenstein, A. Bertini, J. Dennis, C. Deser, C. Fischer, B. Fox-Kemper, J. E. Kay, D. Kinnison, P. J. Kushner, V. E. Larson, M. C. Long, S. Mickelson, J. K. Moore, E. Nienhouse, L. Polvani, P. J. Rasch, W. G. Strand, The community earth system model version 2 (CESM2). *J. Adv. Model. Earth Syst.* **12**, e2019MS001916 (2020).

37. C. Deser, A. Phillips, V. Bourdette, H. Teng, Uncertainty in climate change projections: The role of internal variability. *Clim. Dyn.* **38**, 527–546 (2012).

38. E. Erfani, N. J. Burls, The strength of low-cloud feedbacks and tropical climate: A CESM sensitivity study. *J. Clim.* **32**, 2497–2516 (2019).

39. F.-F. Jin, H.-C. Chen, S. Zhao, M. Hayashi, C. Karamperidou, M. F. Stuecker, R. Xie, L. Geng, "Simple ENSO models" in *El Niño Southern Oscillation in a Changing Climate*, M. J. McPhaden, A. Santoso, W. Cai, Eds. (American Geophysical Union, 2021), pp. 119–151.

40. S. C. Sherwood, M. J. Webb, J. D. Annan, K. C. Armour, P. M. Forster, J. C. Hargreaves, G. Hegerl, S. A. Klein, K. D. Marvel, E. J. Rohling, M. Watanabe, T. Andrews, P. Braconnot, C. S. Bretherton, G. L. Foster, Z. Hausfather, A. S. von der Heydt, R. Knutti, T. Mauritsen, J. R. Norris, C. Proistosescu, M. Rugenstein, G. A. Schmidt, K. B. Tokarska, M. D. Zelinka, An assessment of Earth's climate sensitivity using multiple lines of evidence. *Rev. Geophys.* **58**, e2019RG000478 (2020).

41. R. Knutti, M. A. Rugenstein, G. C. Hegerl, Beyond equilibrium climate sensitivity. *Nat. Geosci.* **10**, 727–736 (2017).

42. T. A. Myers, R. C. Scott, M. D. Zelinka, S. A. Klein, J. R. Norris, P. M. Caldwell, Observational constraints on low cloud feedback reduce uncertainty of climate sensitivity. *Nat. Clim. Change* **11**, 501–507 (2021).

43. Y. Dong, K. C. Armour, C. Proistosescu, T. Andrews, D. S. Battisti, P. M. Forster, D. Paynter, C. J. Smith, H. Shiogama, Biased estimates of equilibrium climate sensitivity and transient climate response derived from historical CMIP6 simulations. *Geophys. Res. Lett.* **48**, e2021GL095778 (2021).

44. P. Forster, T. Storelvmo, K. Armour, W. Collins, J.-L. Dufresne, D. Frame, D. J. Lunt, T. Mauritsen, M.D. Palmer, M. Watanabe, M. Wild, "The Earth's energy budget, climate feedbacks, and climate sensitivity" in *Climate Change 2021: The Physical Science Basis. Contribution of Working Group I to the Sixth Assessment Report of the Intergovernmental Panel on Climate Change*, V. Masson-Delmotte, P. Zhai, A. Pirani, S. L. Connors, C. Péan, S. Berger, N. Caud, Y. Chen, L. Goldfarb, M. I. Gomis, M. Huang, K. Leitzell, E. Lonnoy, J. B. R. Matthews, T. K. Maycock, T. Waterfield, O. Yelekçi, R. Yu, B. Zhou, Eds. (Cambridge Univ. Press, 2021), pp. 923–1054.

45. J. M. Gregory, W. J. Ingram, M. A. Palmer, G. S. Jones, P. A. Stott, R. B. Thorpe, J. A. Lowe, T. C. Johns, K. D. Williams, A new method for diagnosing radiative forcing and climate sensitivity. *Geophys. Res. Lett.* **31**, L03205 (2004).

46. A. Dessler, P. M. Forster, An estimate of equilibrium climate sensitivity from interannual variability. *J. Geophys. Res. Atmos.* **123**, 8634–8645 (2018).

47. M. Huber, U. Beyerle, R. Knutti, Estimating climate sensitivity and future temperature in the presence of natural climate variability. *Geophys. Res. Lett.* **41**, 2086–2092 (2014).

48. Y. Dong, C. Proistosescu, K. C. Armour, D. S. Battisti, Attributing historical and future evolution of radiative feedbacks to regional warming patterns using a Green's function approach: The preeminence of the western Pacific. *J. Clim.* **32**, 5471–5491 (2019).

49. T. Andrews, M. J. Webb, The dependence of global cloud and lapse rate feedbacks on the spatial structure of tropical Pacific warming. *J. Clim.* **31**, 641–654 (2018).

50. J.-Y. Zhuo, C. Y. Lee, A. Sobel, R. Seager, S. J. Camargo, Y. H. Lin, B. Fosu, K. A. Reed, A more La Niña–like response to radiative forcing after flux adjustment in CESM2. *J. Clim.* **38**, 1037–1050 (2025).

51. B. C. O'Neill, C. Tebaldi, D. P. van Vuuren, V. Eyring, P. Friedlingstein, G. Hurtt, R. Knutti, E. Kriegler, J. F. Lamarque, J. Lowe, G. A. Meehl, R. Moss, K. Riahi, B. M. Sanderson, The scenario model intercomparison project (ScenarioMIP) for CMIP6. *Geosci. Model Dev.* **9**, 3461–3482 (2016).

52. L. C. Slivinski, G. P. Compo, J. S. Whitaker, P. D. Sardeshmukh, B. S. Giese, C. McColl, R. Allan, X. Yin, R. Vose, H. Titchner, J. Kennedy, L. J. Spencer, L. Ashcroft, S. Brönnimann, M. Brunet, D. Camuffo, R. Cornes, T. A. Cram, R. Crouthamel, F. Domínguez-Castro, J. E. Freeman, J. Gergis, E. Hawkins, P. D. Jones, S. Jourdain, A. Kaplan, H. Kubota, F. L. Blancq, T. C. Lee, A. Lorrey, J. Luterbacher, M. Maugeri, C. J. Mock, G. W. K. Moore, R. Przybylak, C. Pudmenzky, C. Reason, V. C. Slonosky, C. A. Smith, B. Tinz, B. Trewin, M. A. Valente, X. L. Wang, C. Wilkinson, K. Wood, P. Wyszyński, Towards a more reliable historical

reanalysis: Improvements for version 3 of the twentieth century reanalysis system. *Q. J. Roy. Meteorol. Soc.* **145**, 2876–2908 (2019).

53. H. Hersbach, B. Bell, P. Berrisford, S. Hirahara, A. Horányi, J. Muñoz-Sabater, J. Nicolas, C. Peubey, R. Radu, D. Schepers, A. Simmons, C. Soci, S. Abdalla, X. Abellan, G. Balsamo, P. Bechtold, G. Biavati, J. Bidlot, M. Bonavita, G. de Chiara, P. Dahlgren, D. Dee, M. Diamantakis, R. Dragani, J. Flemming, R. Forbes, M. Fuentes, A. Geer, L. Haimberger, S. Healy, R. J. Hogan, E. Hólm, M. Janisková, S. Keeley, P. Laloyaux, P. Lopez, C. Lupu, G. Radnoti, P. de Rosnay, I. Rozum, F. Vamborg, S. Villaume, J. N. Thépaut, The ERA5 global reanalysis. *Q. J. Roy. Meteorol. Soc.* **146**, 1999–2049 (2020).

54. H. Zuo, M. A. Balmaseda, S. Tietsche, K. Mogensen, M. Mayer, The ECMWF operational ensemble reanalysis–analysis system for ocean and sea ice: A description of the system and assessment. *Ocean Sci.* **15**, 779–808 (2019).

55. M. J. McPhaden, R. Lumpkin, S. Wa, The global tropical moored buoy array. *Proc. OceanObs* **9**, 668–682 (2010).

56. J. Stuivenvolt-Allen, A. Fedorov, Wind-stress perturbation experiments in CESM2, Zenodo (2025); https://doi.org/10.5281/zenodo.16942897.

57. D. Bi, M. Dix, S. Marsland, S. O'Farrell, A. Sullivan, R. Bodman, R. Law, I. Harman, J. Srbinovsky, H. A. Rashid, P. Dobrohotoff, C. Mackallah, H. Yan, A. Hirst, A. Savita, F. B. Dias, M. Woodhouse, R. Fiedler, A. Heerdegen, Configuration and spin-up of ACCESS-CM2, the new generation Australian Community Climate and Earth System Simulator coupled model. *J. South Hemisph. Earth Syst. Sci.* **70**, 225–251 (2020).

58. T. Ziehn, M. A. Chamberlain, R. M. Law, A. Lenton, R. W. Bodman, M. Dix, L. Stevens, Y. P. Wang, J. Srbinovsky, The Australian Earth System Model: ACCESS-ESM1.5. *J. South Hemisph. Earth Syst. Sci.* **70**, 193–214 (2020).

59. T. Wu, Y. Lu, Y. Fang, X. Xin, L. Li, W. Li, W. Jie, J. Zhang, Y. Liu, L. Zhang, F. Zhang, Y. Zhang, F. Wu, J. Li, M. Chu, Z. Wang, X. Shi, X. Liu, M. Wei, A. Huang, Y. Zhang, X. Liu,

The Beijing Climate Center Climate System Model (BCC-CSM): The main progress from CMIP5 to CMIP6. *Geosci. Model Dev.* **12**, 1573–1600 (2019).

60. T. Wu, F. Zhang, J. Zhang, W. Jie, Y. Zhang, F. Wu, L. Li, J. Yan, X. Liu, X. Lu, H. Tan, L. Zhang, J. Wang, A. Hu, BCC-ESM1: Model description and evaluation of aerosol simulations. *Geosci. Model Dev.* **13**, 977–1005 (2020).

61. R. Séférian, P. Nabat, M. Michou, D. Saint-Martin, A. Voldoire, J. Colin, B. Decharme, C. Delire, S. Berthet, M. Chevallier, S. Sénési, L. Franchisteguy, J. Vial, M. Mallet, E. Joetzjer, O. Geoffroy, J. F. Guérémy, M. P. Moine, R. Msadek, A. Ribes, M. Rocher, R. Roehrig, D. Salas-y-Mélia, E. Sanchez, L. Terray, S. Valcke, R. Waldman, O. Aumont, L. Bopp, J. Deshayes, C. Éthé, G. Madec, Evaluation of CNRM Earth System Model, CNRM-ESM2-1: Role of Earth system processes in present-day and future climate. *J. Adv. Model. Earth Syst.* **11**, 4182–4227 (2019).

62. N. C. Swart, J. N. S. Cole, V. V. Kharin, M. Lazare, J. F. Scinocca, N. P. Gillett, J. Anstey, V. Arora, J. R. Christian, S. Hanna, Y. Jiao, W. G. Lee, F. Majaess, O. A. Saenko, C. Seiler, C. Seinen, A. Shao, M. Sigmond, L. Solheim, K. von Salzen, D. Yang, B. Winter, Canadian Earth System Model version 5 (CanESM5.0.3). *Geosci. Model Dev.* **12**, 4823–4873 (2019).

63. J.-C. Golaz, P. M. Caldwell, L. P. van Roekel, M. R. Petersen, Q. Tang, J. D. Wolfe, G. Abeshu, V. Anantharaj, X. S. Asay-Davis, D. C. Bader, S. A. Baldwin, G. Bisht, P. A. Bogenschutz, M. Branstetter, M. A. Brunke, S. R. Brus, S. M. Burrows, P. J. Cameron-Smith, A. S. Donahue, M. Deakin, R. C. Easter, K. J. Evans, Y. Feng, M. Flanner, J. G. Foucar, J. G. Fyke, B. M. Griffin, C. Hannay, B. E. Harrop, M. J. Hoffman, E. C. Hunke, R. L. Jacob, D. W. Jacobsen, N. Jeffery, P. W. Jones, N. D. Keen, S. A. Klein, V. E. Larson, L. R. Leung, H. Y. Li, W. Lin, W. H. Lipscomb, P. L. Ma, S. Mahajan, M. E. Maltrud, A. Mametjanov, J. L. McClean, R. B. McCoy, R. B. Neale, S. F. Price, Y. Qian, P. J. Rasch, J. E. J. Reeves Eyre, W. J. Riley, T. D. Ringler, A. F. Roberts, E. L. Roesler, A. G. Salinger, Z. Shaheen, X. Shi, B. Singh, J. Tang, M. A. Taylor, P. E. Thornton, A. K. Turner, M. Veneziani, H. Wan, H. Wang, S. Wang, D. N. Williams, P. J. Wolfram, P. H. Worley, S. Xie, Y. Yang, J. H. Yoon, M. D. Zelinka, C. S. Zender, X. Zeng, C. Zhang, K. Zhang, Y. Zhang, X. Zheng, T. Zhou, Q. Zhu, The

DOE E3SM coupled model version 1: Overview and evaluation at standard resolution. *J. Adv. Model. Earth Syst.* **11**, 2089–2129 (2019).

64. R. Döscher, M. Acosta, A. Alessandri, P. Anthoni, T. Arsouze, T. Bergman, R. Bernardello, S. Boussetta, L. P. Caron, G. Carver, M. Castrillo, F. Catalano, I. Cvijanovic, P. Davini, E. Dekker, F. J. Doblas-Reyes, D. Docquier, P. Echevarria, U. Fladrich, R. Fuentes-Franco, M. Gröger, J. v. Hardenberg, J. Hieronymus, M. P. Karami, J. P. Keskinen, T. Koenigk, R. Makkonen, F. Massonnet, M. Ménégoz, P. A. Miller, E. Moreno-Chamarro, L. Nieradzik, T. van Noije, P. Nolan, D. O'Donnell, P. Ollinaho, G. van den Oord, P. Ortega, O. T. Prims, A. Ramos, T. Reerink, C. Rousset, Y. Ruprich-Robert, P. le Sager, T. Schmith, R. Schrödner, F. Serva, V. Sicardi, M. Sloth Madsen, B. Smith, T. Tian, E. Tourigny, P. Uotila, M. Vancoppenolle, S. Wang, D. Wårlind, U. Willén, K. Wyser, S. Yang, X. Yepes-Arbós, Q. Zhang, The EC-Earth3 Earth system model for the Coupled Model Intercomparison Project 6. *Geosci. Model Dev.* **15**, 2973–3020 (2022).

65. I. M. Held, H. Guo, A. Adcroft, J. P. Dunne, L. W. Horowitz, J. Krasting, E. Shevliakova, M. Winton, M. Zhao, M. Bushuk, A. T. Wittenberg, B. Wyman, B. Xiang, R. Zhang, W. Anderson, V. Balaji, L. Donner, K. Dunne, J. Durachta, P. P. G. Gauthier, P. Ginoux, J. C. Golaz, S. M. Griffies, R. Hallberg, L. Harris, M. Harrison, W. Hurlin, J. John, P. Lin, S. J. Lin, S. Malyshev, R. Menzel, P. C. D. Milly, Y. Ming, V. Naik, D. Paynter, F. Paulot, V. Ramaswamy, B. Reichl, T. Robinson, A. Rosati, C. Seman, L. G. Silvers, S. Underwood, N. Zadeh, Structure and performance of GFDL's CM4.0 climate model. *J. Adv. Model. Earth Syst.* **11**, 3691–3727 (2019).

66. J. P. Dunne, L. W. Horowitz, A. J. Adcroft, P. Ginoux, I. M. Held, J. G. John, J. P. Krasting, S. Malyshev, V. Naik, F. Paulot, E. Shevliakova, C. A. Stock, N. Zadeh, V. Balaji, C. Blanton, K. A. Dunne, C. Dupuis, J. Durachta, R. Dussin, P. P. G. Gauthier, S. M. Griffies, H. Guo, R. W. Hallberg, M. Harrison, J. He, W. Hurlin, C. McHugh, R. Menzel, P. C. D. Milly, S. Nikonov, D. J. Paynter, J. Ploshay, A. Radhakrishnan, K. Rand, B. G. Reichl, T. Robinson, D. M. Schwarzkopf, L. T. Sentman, S. Underwood, H. Vahlenkamp, M. Winton, A. T. Wittenberg, B. Wyman, Y. Zeng, M. Zhao, The GFDL Earth System Model Version 4.1 (GFDL-ESM 4.1): Overall coupled model description and simulation characteristics. *J. Adv. Model. Earth Syst.* **12**, e2019MS002015 (2020).

67. M. Kelley, G. A. Schmidt, L. S. Nazarenko, S. E. Bauer, R. Ruedy, G. L. Russell, A. S. Ackerman, I. Aleinov, M. Bauer, R. Bleck, V. Canuto, G. Cesana, Y. Cheng, T. L. Clune, B. I. Cook, C. A. Cruz, A. D. Del Genio, G. S. Elsaesser, G. Faluvegi, N. Y. Kiang, D. Kim, A. A. Lacis, A. Leboissetier, A. N. Le Grande, K. K. Lo, J. Marshall, E. E. Matthews, S. M. Dermid, K. Mezuman, R. L. Miller, L. T. Murray, V. Oinas, C. Orbe, C. P. García-Pando, J. P. Perlwitz, M. J. Puma, D. Rind, A. Romanou, D. T. Shindell, S. Sun, N. Tausnev, K. Tsigaridis, G. Tselioudis, E. Weng, J. Wu, M.-S. Yao, GISS-E2.1: Configurations and climatology. *J. Adv. Model. Earth Syst.* **12**, e2019MS002025 (2020).

68. K. D. Williams, D. Copsey, E. W. Blockley, A. Bodas-Salcedo, D. Calvert, R. Comer, P. Davis, T. Graham, H. T. Hewitt, R. Hill, P. Hyder, S. Ineson, T. C. Johns, A. B. Keen, R. W. Lee, A. Megann, S. F. Milton, J. G. L. Rae, M. J. Roberts, A. A. Scaife, R. Schiemann, D. Storkey, L. Thorpe, I. G. Watterson, D. N. Walters, A. West, R. A. Wood, T. Woollings, P. K. Xavier, The met office global coupled model 3.0 and 3.1 (GC3.0 and GC3.1) configurations. *J. Adv. Model. Earth Syst.* **10**, 357–380 (2018).

69. E. M. Volodin, E. V. Mortikov, S. V. Kostrykin, V. Y. Galin, V. N. Lykossov, A. S. Gritsun, N. A. Diansky, A. V. Gusev, N. G. Iakovlev, A. A. Shestakova, S. V. Emelina, Simulation of the modern climate using the INM-CM48 climate model. *Russ. J. Numer. Anal. Math. Model.* **33**, 367–374 (2018).

70. E. M. Volodin, E. V. Mortikov, S. V. Kostrykin, V. Y. Galin, V. N. Lykossov, A. S. Gritsun, N. A. Diansky, A. V. Gusev, N. G. Iakovlev, Simulation of the present-day climate with the climate model INMCM5. *Clim. Dyn.* **49**, 3715–3734 (2017).

71. T. Hajima, M. Watanabe, A. Yamamoto, H. Tatebe, M. A. Noguchi, M. Abe, R. Ohgaito, A. Ito, D. Yamazaki, H. Okajima, A. Ito, K. Takata, K. Ogochi, S. Watanabe, M. Kawamiya, Development of the MIROC-ES2L Earth system model and the evaluation of biogeochemical processes and feedbacks. *Geosci. Model Dev.* **13**, 2197–2244 (2020).

72. H. Tatebe, T. Ogura, T. Nitta, Y. Komuro, K. Ogochi, T. Takemura, K. Sudo, M. Sekiguchi, M. Abe, F. Saito, M. Chikira, S. Watanabe, M. Mori, N. Hirota, Y. Kawatani, T. Mochizuki, K. Yoshimura, K. Takata, R. O'ishi, D. Yamazaki, T. Suzuki, M. Kurogi, T. Kataoka, M.

Watanabe, M. Kimoto, Description and basic evaluation of simulated mean state, internal variability, and climate sensitivity in MIROC6. *Geosci. Model Dev.* **12**, 2727–2765 (2019).

73. J. Cao, B. Wang, Y. M. Yang, L. Ma, J. Li, B. Sun, Y. Bao, J. He, X. Zhou, L. Wu, The NUIST Earth System Model (NESM) version 3: Description and preliminary evaluation. *Geosci. Model Dev.* **11**, 2975–2993 (2018).

74. S. Park, J. Shin, S. Kim, E. Oh, Y. Kim, Global climate simulated by the Seoul National University Atmosphere Model version 0 with a Unified Convection Scheme (SAM0-UNICON). *J. Clim.* **32**, 2917–2949 (2019).

75. A. A. Sellar, C. G. Jones, J. P. Mulcahy, Y. Tang, A. Yool, A. Wiltshire, F. M. O'Connor, M. Stringer, R. Hill, J. Palmieri, S. Woodward, L. de Mora, T. Kuhlbrodt, S. T. Rumbold, D. I. Kelley, R. Ellis, C. E. Johnson, J. Walton, N. L. Abraham, M. B. Andrews, T. Andrews, A. T. Archibald, S. Berthou, E. Burke, E. Blockley, K. Carslaw, M. Dalvi, J. Edwards, G. A. Folberth, N. Gedney, P. T. Griffiths, A. B. Harper, M. A. Hendry, A. J. Hewitt, B. Johnson, A. Jones, C. D. Jones, J. Keeble, S. Liddicoat, O. Morgenstern, R. J. Parker, V. Predoi, E. Robertson, A. Siahaan, R. S. Smith, R. Swaminathan, M. T. Woodhouse, G. Zeng, M. Zerroukat, UKESM1: Description and evaluation of the U.K. Earth System Model. *J. Adv. Model. Earth Syst.* **11**, 4513–4558 (2019).

76. A. Voldoire, D. Saint-Martin, S. Sénési, B. Decharme, A. Alias, M. Chevallier, J. Colin, J.-F. Guérémy, M. Michou, M.-P. Moine, P. Nabat, R. Roehrig, D. Salas y Mélia, R. Séférian, S. Valcke, I. Beau, S. Belamari, S. Berthet, C. Cassou, J. Cattiaux, J. Deshayes, H. Douville, C. Ethé, L. Franchistéguy, O. Geoffroy, C. Lévy, G. Madec, Y. Meurdesoif, R. Msadek, A. Ribes, E. Sanchez-Gomez, L. Terray, R. Waldman, L. Terray, Evaluation of CMIP6 DECK experiments with CNRM-CM6-1. *J. Adv. Model. Earth Syst*. **11**, 2177–2213 (2019).

77. O. Boucher, J. Servonnat, A. L. Albright, O. Aumont, Y. Balkanski, V. Bastrikov, S. Bekki, R. Bonnet, S. Bony, L. Bopp, P. Braconnot, P. Brockmann, P. Cadule, A. Caubel, F. Cheruy, F. Codron, A. Cozic, D. Cugnet, F. D'Andrea, P. Davini, C. de Lavergne, S. Denvil, J. Deshayes, M. Devilliers, A. Ducharne, J.-L. Dufresne, E. Dupont, C. Éthé, L. Fairhead, L. Falletti, S. Flavoni, M.-A. Foujols, S. Gardoll, G. Gastineau, J. Ghattas, J.-Y. Grandpeix, B. Guenet, L.E. Guez, E. Guilyardi, M. Guimberteau, D. Hauglustaine, F. Hourdin, A. Idelkadi,

S. Joussaume, M. Kageyama, M. Khodri, G. Krinner, N. Lebas, G. Levavasseur, C. Lévy, L. Li, F. Lott, T. Lurton, S. Luyssaert, G. Madec, J.-B. Madeleine, F. Maignan, M. Marchand, O. Marti, L. Mellul, Y. Meurdesoif, J. Mignot, I. Musat, C. Ottlé, P. Peylin, Y. Planton, J. Polcher, C. Rio, N. Rochetin, C. Rousset, P. Sepulchre, A. Sima, D. Swingedouw, R. Thiéblemont, A. K. Traore, M. Vancoppenolle, J. Vial, J. Vialard, N. Viovy, N. Vuichard, Presentation and evaluation of the IPSL-CM6A-LR climate model. *J. Adv. Model. Earth Syst.* **12**, e2019MS002010 (2020).

78. Yukimoto, H. Kawai, T. Koshiro, N. Oshima, K. Yoshida, S. Urakawa, H. Tsujino, M. Deushi, T. Tanaka, M. Hosaka, S. Yabu, H. Yoshimura, E. Shindo, R. Mizuta, A. Obata, Y. Adachi, M. Ishii, The Meteorological Research Institute Earth System Model Version 2.0, MRI-ESM2.0: Description and basic evaluation of the physical component. *J. Meteorol. Soc. Jpn.* **97**, 931–965 (2019).
